# Molecular Characterization, Tissue Distribution and Differential Nutritional Regulation of Three n-3 LC-PUFA Biosynthesis-Related Genes in Hybrid Grouper (*Epinephelus fuscoguttatus* ♀ × *Epinephelus lanceolatus* ♂)

**DOI:** 10.3390/ani12030234

**Published:** 2022-01-19

**Authors:** Qingjun Wu, Zhi Zheng, Chuijin Wang, Yao Wang, Yuejia Sun, Yujie Gao

**Affiliations:** College of Marine Science, Hainan University, Haikou 570228, China; 19090800210014@hainanu.edu.cn (Q.W.); 18090800210019@hainanu.edu.cn (Z.Z.); wangchuijin888@163.com (C.W.); 18152235096@163.com (Y.W.); 19095134210029@hainanu.edu.cn (Y.S.)

**Keywords:** *Serranidae*, cross-breeding, synthesis pathway, RACE cloning, differentially expressed gene

## Abstract

**Simple Summary:**

Polyunsaturated fatty acids, especially DHA and EPA, play crucial roles in fish growth performance, brain and eye development, reproduction and non-specific immune responses. However, considering the environmental unsustainability and the increasing price of fisheries’ by-products, alternative aquafeed ingredients are needed as a source of unsaturated fatty acids for farming marine fish. Here, we isolated and characterized three genes participating in the biosynthesis of n-3 LC-PUFA in hybrid groupers. We found that these genes were expressed in the liver of hybrid groupers in response to dietary fatty acid levels. Our findings contribute to a better understanding of the n-3 LC-PUFA biosynthesis process in this marine fish species.

**Abstract:**

Elongases of very long-chain fatty acids (Elovls) and fatty acid desaturases (Fads) are crucial enzymes involved in the biosynthesis of long-chain polyunsaturated fatty acids (LC-PUFAs). In this paper, we report the molecular cloning and characterization of three genes from the marine teleost *Epinephelus fuscoguttatus* ♀ × *Epinephelus lanceolatus* ♂, and analyzed tissue distribution and their expression in response to dietary n-3 LC-PUFA levels after a 42-day feeding experiment. The *elovl5*, *elovl8* and *fads2* genes encoded 294, 263 and 445 amino acids, respectively, which exhibited all the characteristics of the Elovl and Fads family. Tissue distribution analysis revealed that *elovl5*, *elovl8* and *fads2* were widely transcribed in various tissues, with the highest level in the brain, as described in other carnivorous marine teleosts. The transcript levels of *elovl5*, *elovl8* and *fads2* in the liver were significantly affected by dietary n-3 LC-PUFA, and higher LC-PUFA levels repressed their expression. These results demonstrated, for the first time, the presence and nutritional modulation of *elovl5*, *elovl8* and *fads2* cDNA in the juvenile hybrid grouper. Further studies are needed to determine the functional characterization of these genes and explore the mechanism of these genes when regulated by dietary fatty lipid profiles in this species.

## 1. Introduction

Long-chain polyunsaturated fatty acids (LC-PUFA, ≥C20 and ≥2 double bonds) are widely present in organisms and play vital roles in maintaining cell membrane fluidity, regulating fat metabolism, enhancing immunity and reducing inflammation [1]. Among these, eicosapentaenoic acid (EPA) and docosahexaenoic acid (DHA) are the most critical LC-PUFAs, with beneficial effects on human health particularly in the prevention of cardiovascular and neuro developmental disorders [2]. At present, fish, particularly marine species, represent a rich source of EPA and DHA for human consumers, due to the high content of LC-PUFAs acquired from the feed [3]. However, the raw materials for aquaculture feed have reached a sustainable limit, and there is a rapid and continuous growth in aquaculture activities; it is estimated that 75% of the world’s fish oil supplies are used for aquaculture production [4]. Thus, aquaculture nutritionists and aqua-feed companies are seeking sustainable alternatives to replace marine fish oil. However, the other issue is that the alternatives, primarily plant oils rich in C18 PUFA but devoid of LC-PUFA significantly decreased n-3 LC-PUFA in the filet of farmed fish [5,6]. Therefore, there is considerable interest in understanding the n-3 LC-PUFA biosynthesis pathway and nutritional regulation mechanism in the cultured fish species.

LC-PUFA biosynthesis depends on the specific gene repertoire and each enzyme’s respective functional capabilities, which may vary from species to species [7]. Elovls are the initial and rate-limiting enzymes responsible for the elongation reaction required for the *de novo* biosynthesis of LC-PUFA. At present, the Elovls family was found to have Elovl1-8, in which Elovl2, Elovl4, Elovl5 and Elovl8 are involved in the elongation of LC-PUFA [8,9,10]. Elovl2 is preferentially responsible for the elongation step from C22 to C24 LC-PUFA. It has been found in freshwater and anadromous species such as zebrafish (*Danio rerio*), African catfish (*Clarias gariepinus*), and Atlantic salmon (*Salmo salar*), as well in chordates such as sea lamprey (*Petromyzon marinus*, agnathan) and the elephant shark (*Callorhinchus milii*, basal gnathostome) [11,12,13,14]. Thus, the variation between marine and freshwater fish species in the presence or absence of Elovl2, a critical enzyme for DHA synthesis, was hypothesized to contribute to the low ability of most farmed marine fish to biosynthesize DHA [15].

Elovl4 and Elovl5 have been isolated from several marine fish species [16]. Elovl5 can preferentially elongate C18 (18:3n-6 and 18:4n-3) and C20 (20:4n-6 and EPA) fatty acids but has low activity toward C22 fatty acids [17]. Elovl4 can effectively extend C18 PUFAs and participate in the synthesis of DHA. Accordingly, it is expected that this enzyme could compensate for Elovl2 activity in some marine fish species, such as rabbitfish (*Siganus canaliculatus*) [18]. Recently, two Elovl8 isoforms, “Elovl8a” and “Elovl8b”, have been found in zebrafish [9] and rabbitfish [19]. Further functional characterization by heterologous expression in yeast showed that Elovl8b could elongate C18 (18:2n-6, 18:3n-3 and 18:4n-3) and C20 (20:4n-6 and 20:5n-3) polyunsaturated fatty acids (PUFAs) to longer-chain polyunsaturated fatty acids (LC-PUFAs), whereas Elovl8a lacked this ability in rabbitfish. However, studies in CRISPR/Cas9 Elovl8a^−/−^ and Elovl8b^−/−^ zebrafish revealed that Elovl8a activity was specific to C18-C20 PUFAs and Elovl8b activity was specific to C18:0 and C20:1 MUFAs. Fatty acid desaturase enzymes introduce double bonds into specific positions on the carbon chain to produce desaturation and are crucial to the synthesis of DHA and 22:5n-6 from EPA and 22:4n-6, respectively, via the “Sprecher pathway” [20]. The Fads gene family was found to have Fads1-3 [21]. However, in most of the teleost species, only Fads2 was present, and this enzyme was more similar to mammalian Δ6 desaturase in most fish species [22]. Therefore, to compensate for the loss of Fads1, some teleost Fads2 have exhibited additional desaturase activity, such as Δ5 in rainbow trout [23], Δ6/Δ5 and ∆4 in rabbitfish [24], and ∆4 in Senegalese sole (*Solea senegalensis*, 1858) [25].

Grouper aquaculture is an important sector of marine fish production in China, with about 190,000 t being produced yearly [26]. Among these, a hybrid grouper of tiger grouper (*Epinephelus fuscoguttatus* ♀) and giant grouper (*Epinephelus lanceolatus* ♂) have become the dominant cultivated species due to their rapid growth and high disease resistance characters [27]. However, to our knowledge, little information is available regarding the genes involved in LC-PUFA biosynthesis in this fish species. Previous studies in a similar species, the orange-spotted grouper (*Epinephelus coioides*), have suggested that it has a limited ability to synthesize n-3 LC-PUFA, especially DHA [28,29]. Thus, to fully understand the LC-PUFA biosynthetic abilities of this new hybrid grouper, we reported the molecular cloning, phylogenetic analysis and nutritional regulation of *elovl5*, *elovl8* and *fads2* under different dietary LC-PUFA levels in this fish.

## 2. Materials and Methods

### 2.1. Fish and Sampling

Juvenile hybrid groupers (4 months old, approximate 20 g) were selected in our trial as the first few months of life are the most vulnerable period and crucial for fish growth. They were obtained from a local commercial hatchery (Wenchang, Hainan province, China), and were acclimated in circular tanks at room temperature (28 °C) for two days. The fish were provided a natural photoperiod and adequate dissolved oxygen (>6.0 mg L^−1^). Four fish were collected and anesthetized with MS-222 (Sigma, St. Louis, MO, USA); then, tissues including liver, heart, intestine, muscle, kidney, stomach, pyloric cease and brain were snap-frozen by liquid nitrogen and then stored at −80 °C until use.

### 2.2. Gene Cloning 

#### 2.2.1. Primers Design

According to the highly conserved domains of the *elovl5*, *elovl8* and *fads2* gene sequence obtained by DNAssist software (Version 1.0) from other species, including cobia (*Rachycentron canadum*), Atlantic salmon, golden pompano (*Trachinotus ovatus*), orange-spotted grouper and giant grouper in the GenBank database (http://www.ncbi.nlm.nih.gov/genbank/ (accessed on 4 January 2022)), specific PCR primers were designed using Primer 5.0 based on the consensus sequences to amplify and confirm the full length of these cDNAs (Table 1) (Figure S1).

#### 2.2.2. RNA Extraction and cDNA Synthesis

Total RNA was extracted from the liver, heart, intestine, muscle, kidney, stomach, pyloric cease and brain using Trizol Reagent (Invitrogen, Waltham, MA, USA) according to the manufacturer’s instructions. Quantification of RNA was performed using NanoDrop ND-1000 spectrophotometer (Wilmington, DE, USA), and the quality was measured by electrophoresis on a 1.0% denaturing agarose gel. The RNA (1 μg) from all the above tissues was reverse-transcribed into cDNA by PrimeScript™ RT kit and gDNA Eraser (Perfect Real Time) (Takara, Japan) according to the manufacturer’s instructions. To obtain the first partial fragment of gene cloning, RNA (1 μg) from liver samples was reverse-transcribed into cDNA using SMARTer® RACE5′/3′ kit (Takara, Japan) and GoScript™ Reverse Transcription System kit (Promega, USA), according to the manufacturer’s instructions. The first partial fragment was sequenced by Sangon Biotech Co., Ltd (Shanghai, China), and was verified by NCBI BlastN. 

#### 2.2.3. RACE Amplification

The 5′ and 3′ rapid amplification of cDNA ends (RACE) PCR template were obtained according to the manufacturer’s instructions (SMARTer™ RACE cDNA Amplification Kit, Clontech, San Jose, CA USA). For 3′ and 5′ RACE of *elovl8*, gene-specific primers, Elovl8-3F and Elovl8-5R, and Universal Primer (provided in the kit) were used in gradient PCR. Amplification was performed in a total volume of 20 μL, containing 10 μL 2×Taq PCR Master Mix II, 0.5 μL of each primer (10 μM), 7 μL nuclease-free water, and 2 μL cDNA (50 ng/μL). For 3′ and 5′ RACE of *elovl5*, Elovl5-3F and Elovl5-5R, and Universal Primer were used in gradient PCR. For 3′ and 5′ RACE of *fads2*, Fads2-3F1 and Fads2-5R1, and Universal Primer were used in gradient PCR. The PCR product was purified, cloned into a pMD19-T vector (Takara, Japan), then transformed and sequenced. The full lengths of the grouper *elovl5*, *elovl8* and *fads2* cDNA were obtained by aligning the first partial sequence with the corresponding RACE PCR products using DNAssist software (Version 1.0). The cloned PCR fragments were then sequenced by Sangon Biotech Co. Ltd. (Shanghai, China).

#### 2.2.4. Sequence and Phylogenetic Analysis

The nucleotide sequence and the amino acid sequence of Elovl5, Elovl8 and Fads2 were analyzed by the NCBI blast program (http://www.ncbi.nlm.nih.gov/blast/Blast.cgi (accessed on 4 January 2022)). The conserved domains of Elovl5, Elovl8, and Fads2 sequences were predicted by the SMART and CDD programs. The molecular weight, isoelectric point, and amino acid content were identified by online software ExPASy (http://web.expasy.org/protparam/ (accessed on 4 January 2022)). The Elovl5, Elovl8 and Fads2 amino acids’ multiple sequence alignments were found using the CLUSTALX software (Version 1.8.1). The sequences alignment of Elovl4, Elovl5 and Elovl8 were uploaded into MEGA 10.0 and all columns containing 75% gaps were stripped from the alignment, leaving a total of 232 positions for phylogenetic analysis. The sequences alignment of Fads2 were uploaded into MEGA 10.0 and all columns containing 4.3% gaps were stripped from the alignment, leaving a total of 438 positions for phylogenetic analysis. The evolutionary model was determined using the MODELS option in MEGA 10.0 resulting in a LG + G, and branch support was calculated using maximum likelihood (Figure S2). The number of booststrap replications for phylogeny test are 1000.2.2. Feeding trial with different n-3 LC-PUFA levels

Three isoenergetic (339 kcal/100 g), isonitrogenous (53%) and isolipidic (7%) experimental diets (Table 2) were formulated containing graded levels of n-3 LC-PUFA (0.53%, 1.19% and 2.69%) based on our previous study (Table 3) (Figure S3). Juvenile hybrid groupers were obtained from a local commercial hatchery (Wenchang, Hainan province, China). Fish were acclimated with commercial diets for 2 weeks before the experiment. A total of 108 fish (average initial weight, 20.8 ± 0.03 g) were selected and randomly distributed into 9 glass tanks (L 60 cm × W 45 cm × H 50 cm) connected to a water-recycling system. The water was oxygenated through air stones at the bottom of each tank. Triplicate groups of fish were fed to apparent satiation by hand twice daily (8:00 am and 4:30 pm). Water temperature (29 ± 0.5 °C), total ammonia (0–0.15 mg/L) and dissolved oxygen (5.9 ± 0.1 mg/L) were monitored daily. Fish were exposed to a 12 h: 12 h light: dark cycle. The feeding trial lasted 42 days. At the end of the experiment, fish were counted and weighed after being subjected to 16 h fasting. Two fish per tank were randomly collected and anesthetized with MS-222 at 80 mg L^-1^ (Sigma, St. Louis, MO, USA): Liver was sampled and snap-frozen by liquid nitrogen and then stored at −80 °C for the RNA extraction.

### 2.3. Quantitative Real-Time PCR

RT-PCR was carried out in a quantitative thermal cycler (Roche Light Cycler® 480, Switzerland). The amplification was performed in a total volume of 20 μL containing 10 μL power SYBR® Green PCR Master Mix (Takara, Japan), 0.8 μL of each primer (10 μM), 6.4 μL nuclease-free water and 2 μL of cDNA (50 ng/μL). The real-time RT-PCR program was as follows: 95 °C for 30 s, followed by 40 cycles of 95 °C for 5 s, 60 °C for 20 s, and 65 °C for 15 s. After the amplification phase, a melt curve of 0.5 °C increments from 65 °C to 90 °C was performed, enabling the confirmation of the amplification of a single product in each reaction for the melting curve. Standard curves were made with five different dilutions (in triplicate) of the cDNA samples and the amplification efficiency was analyzed according to the following equation E = 10^(−1/slope)^−1. Real-time PCR efficiencies of *elovl8*, *elovl5*, *fads2* and elongation factor 1 (*ef1alpha*) ranged between 96.2% and 104.7%, 96.5% and 105.5%, 97.1% and 106.1%, 95.6% and 106.2%, respectively. *Ef1alpha* was used as the housekeeping gene to normalize of the results (as our previous study). The expression levels of the target genes were calculated followed by the 2−^∆∆Ct^ method. 

### 2.4. Statistical Analysis

The normality and homogeneity of the data were explored using Hartley’s test. Then, data were subjected to one-way analysis of variance (ANOVA) and Duncan’s multiple range test (SPSS 22.0 for Windows, Chicago IL, USA) to determine if significant differences occurred between different treatments. Differences were considered significant at *p* < 0.05. All data are presented as mean ± S.E.M.

## 3. Results

### 3.1. Gene Cloning and Sequence Analysis of elovl5, elovl8 and fads2

The cloned *elovl5* cDNA from the hybrid grouper (uploaded under GenBank Accession Number MZ713365) was 1226 bp, which contained an 885 bp ORF that encoded a polypeptide of 294 amino acids, a 41 bp 5′ UTR and a 300 bp 3′ UTR (Figure S4). The hybrid grouper Elovl5 protein has a calculated molecular mass of 35.10 kDa and an isoelectric point of 9.25 amino acid sequence deduced by SMART analysis, which has 7 transmembrane domain located at residues 32–50, 63–85, 111–132, 139–161, 176–198, 205-227 and 231–250 respectively, a highly conserved redox center histidine cluster (143–147), 3 conserved motifs (120–127, 173–184 and 203–209), and an endoplasmic reticulum retention signal (288–294). The NCBI-CDD program showed that Elovl5 included a conservative region of LC-PUFA elongases located at residues 28–256.

The cloned *elovl8* cDNA from a hybrid grouper (uploaded GenBank Accession Number MZ713364) was 1228 bp, which contained a 792 bp ORF that encoded a polypeptide of 263 amino acids with a 111 bp 5′ UTR and a 325 bp 3′ UTR (Figure S5). The encoded Elovl8 protein has a calculated molecular mass of 30.8 kDa and an isoelectric point of 9.21 amino acid sequence, deduced by SMART analysis. Meanwhile, the Elovl8 protein possessed 7 transmembrane domains located at residues 25–50, 63–85, 111–113, 140–159, 169–191, 203–225 and 105–127, respectively, a highly conserved redox center histidine cluster (259-–263), 4 conserved motifs (121–128, 139–148, 174–185 and 205–211), and an endoplasmic reticulum retention signal (259–263). The NCBI-CDD program showed that the newly cloned Elovl8 possessed a conservative region of LC-PUFA elongases located at residues 28–262.

The cloned *fads2* cDNA from the hybrid grouper (uploaded under GenBank Accession Number MZ713366) was 2006 bp, which contained a 1338 bp ORF that encoded a polypeptide of 445 amino acids, a 238 bp 5′ UTR and a 430 bp 3′ UTR (Figure S6). The encoded Fads2 protein has a calculated molecular mass of 52.04 kDa and isoelectric point of 8.41 amino acid sequence deduced by SMART analysis, which had 3 transmembrane domain located at residues 148–170, 267–289 and 304–326, respectively, 3 highly conserved redox center histidine cluster (181-185, 218-222 and 383-387), 3 conserved motifs (120–127, 173–184 and 203–209), and an N-terminal Cyt-b5 domain with typical heme-binding motif (288–294). The NCBI-CDD program showed that Fad2 included a conservative region located at residues 163–413.

### 3.2. Multiple Sequence Alignment and Phylogenetic Analysis

**Figure 1:**
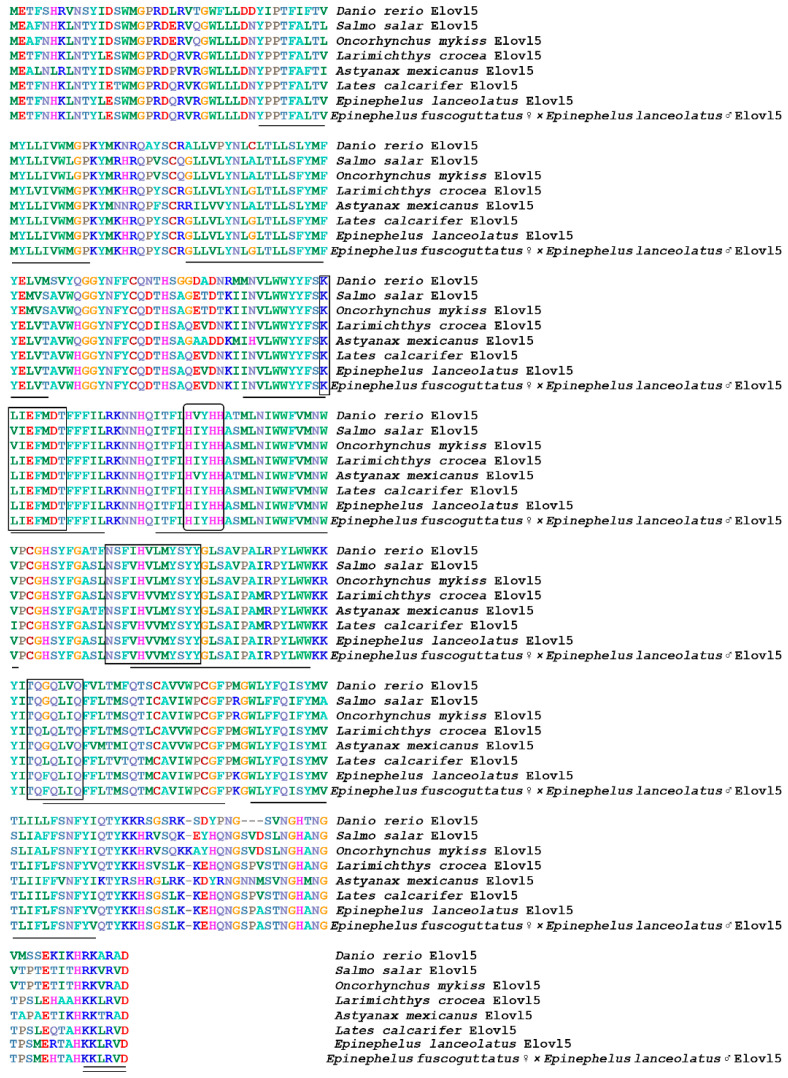
Multiple amino acids sequence alignment of Elovl5 from different fish species. The transmembrane region of amino acids is represented by a straight line; the conserved motif is marked with a square; the histidine box is marked with an ellipse, and endoplasmic reticulum signal is marked with a double line.

**Figure 2:**
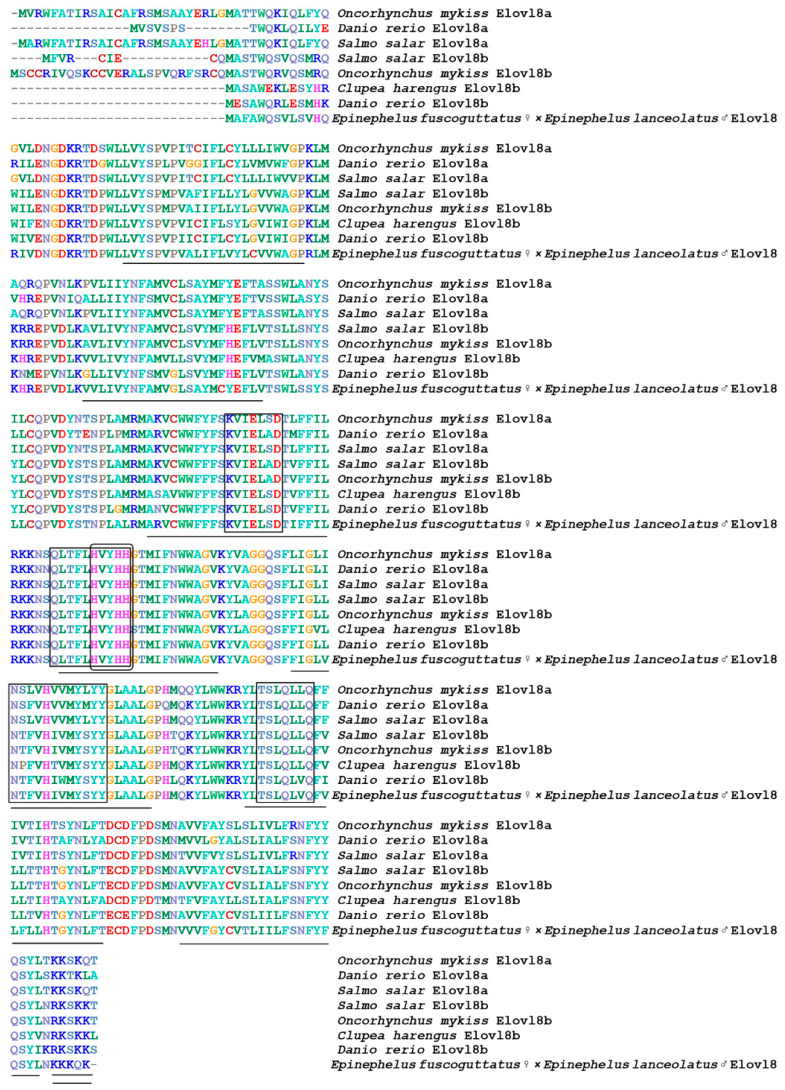
Multiple amino acids sequence alignment of Elovl8 from different species. The transmembrane region of amino acids is represented by a straight line; the conserved motif is marked with a square; the histidine box is marked with an ellipse, and endoplasmic reticulum signal is marked with a double line.

**Figure 3:**
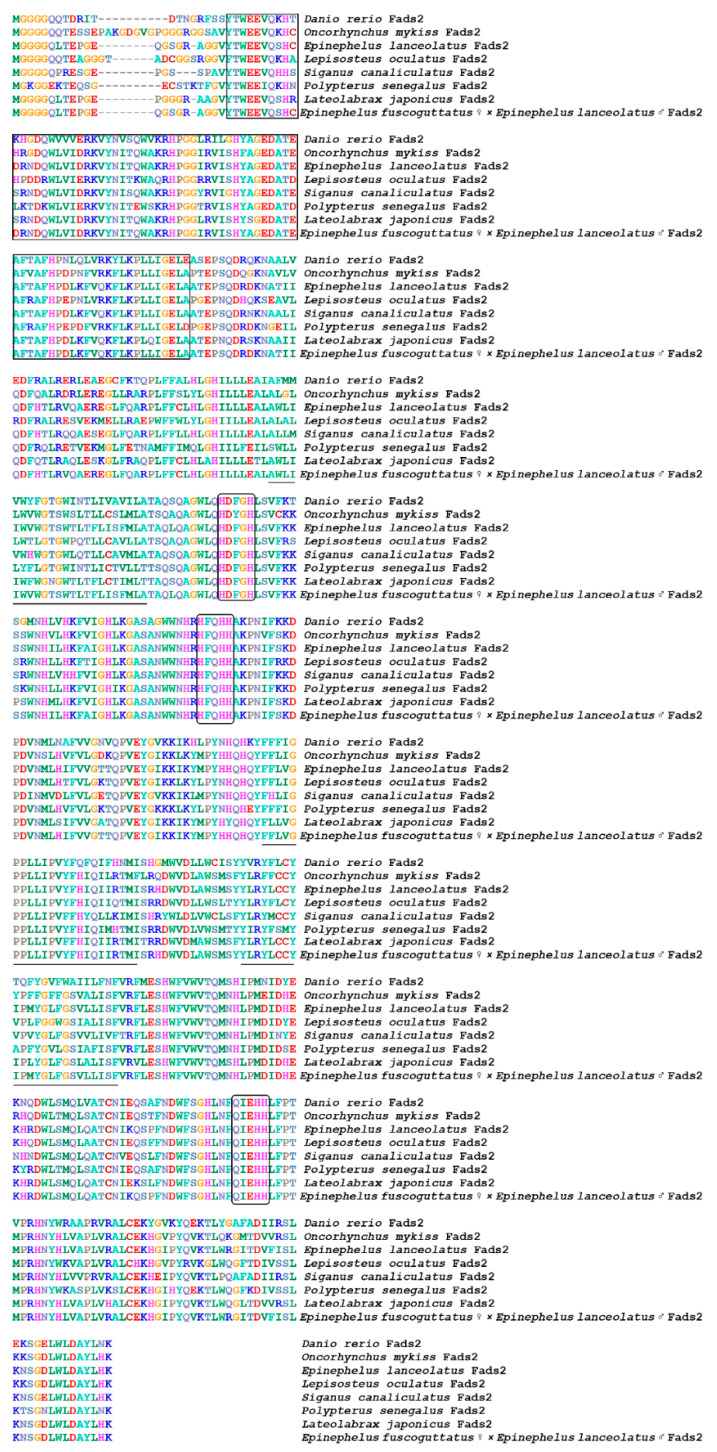
Multiple amino acids sequence alignment of Fads2 from different species. The transmembrane region of amino acids is represented by a straight line; the conserved motif is marked with a square; the histidine box is marked with an ellipse, and endoplasmic reticulum signal is marked with a double line.

A multiple sequence alignment and phylogenetic analysis were conducted to investigate the homology of genes *elovl5*, *elovl8 and fads2* between hybrid grouper and other species. The results showed that most of the amino acid residues in fish, including hybrid grouper, were highly aligned with Elovl5 and Elovl8 (Figure 1 and Figure 2). They possessed all the characteristic features of Elovl family members, including multiple transmembrane regions, a single-histidine dideoxy-binding motif HXXHH, the putative endoplasmic reticulum (ER) retrieval signal KKKQK and KKLRVD, respectively, at the carboxyl terminus and multiple regions, containing similar motifs, such as (1) KXXEXXDT, (2) QXXFLHXXHH, (3) NXXXHXXMYXYY, (4) and TXXQXXQ.Fads2 possessed all the characteristic features of Fads family members, including multiple transmembrane regions, and three single-histidine dideoxy-binding motifs: HXXHH, HDXGH and QXXHH (Figure 3).

**Figure 4:**
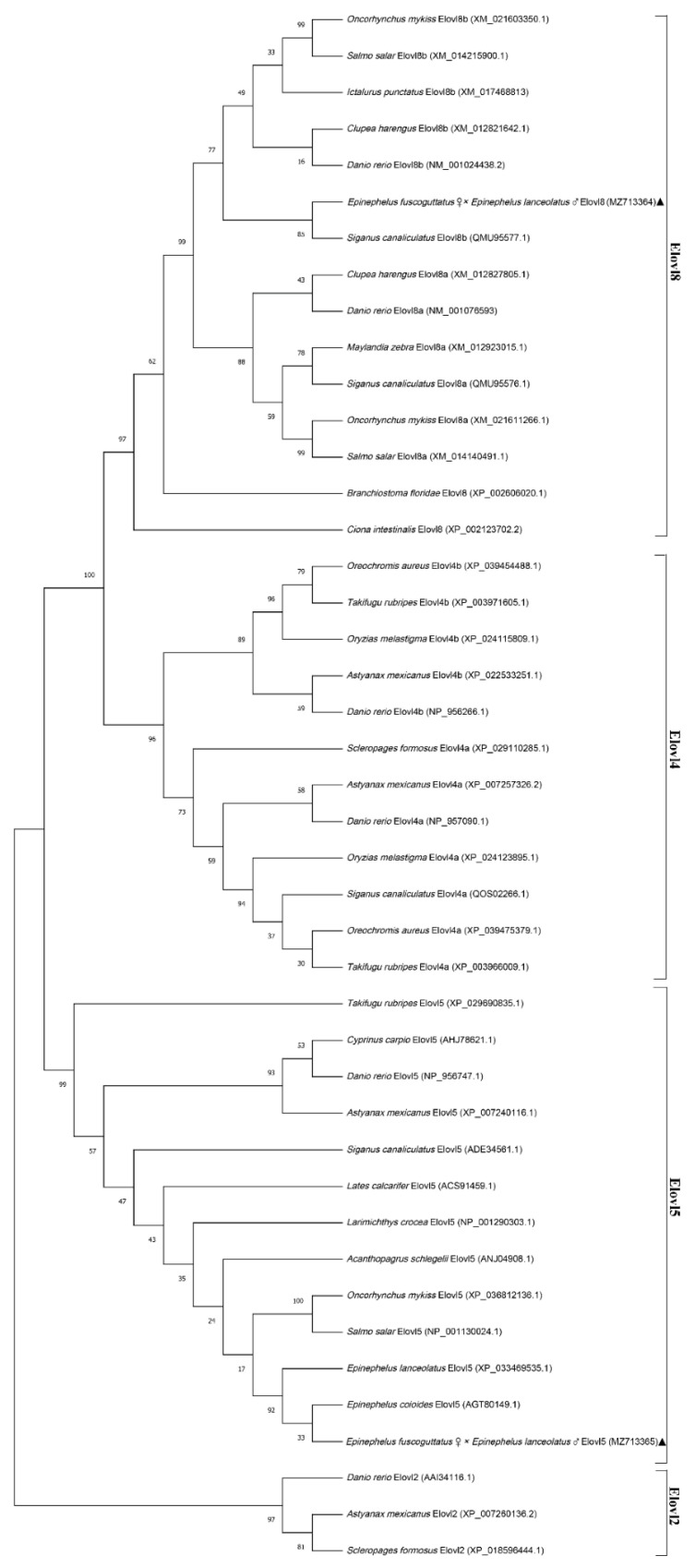
Phylogenetic tree analysis constructed by using the full-length amino acid sequences of Elovl8, Elovl4 and Elovl5. The full-length amino acid sequence of Elovl8, Elovl4 and Elovl5 proteins were extracted from Genbank and analyzed using the maximum likelihood method by Mega x with 1000 bootstrap replications. Elovl2 is regarded as the outgroup. The numbers shown at branches indicated the bootstrap values (%). Our *Epinephelus fuscoguttatus* ♀ × *Epinephelus lanceolatus* ♂ sequence was marked “▲”.

Phylogenetic analysis by the maximum likelihood method showed three clusters for elovl8, elovl4 and elovl5, with elovl8 cluster showing closest relationship to the elovl4 cluster (Figure 4). Amino acid sequence of hybrid grouper Elovl8 exhibited high identity with *Siganus_canaliculatus* Elovl8b (86%), *Salmo salar* Elovl8b (83%) and *Danio rerio* Elovl8b (80%). Elovl5 was closest to Perciformes, such as *Epinephelus coioides* (99%), *Epinephelus lanceolatus* (99%), *Acanthopagrus schlegelii* (95%) and *Larimichthys crocea* (95%).

**Figure 5:**
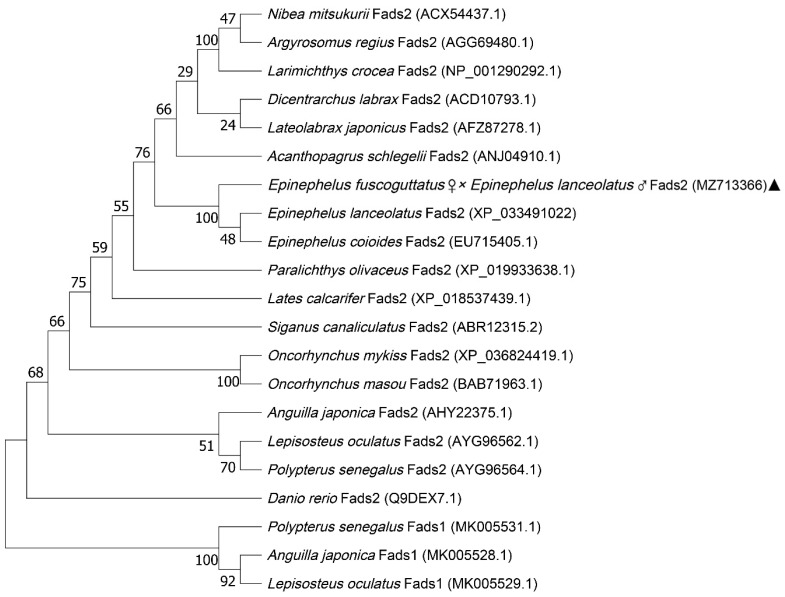
Phylogenetic tree analysis constructed by using full-length amino acid sequences of Fads2. The full-length amino acid sequence of Fads2 and Fads1 proteins were extracted from Genbank and analyzed using Maximum Likelihood method by Mega x with 1000 bootstrap replications. The numbers shown at branches indicated the bootstrap values (%). Fads1 is regarded as the outgroup. Our *Epinephelus fuscoguttatus* ♀ × *Epinephelus lanceolatus* ♂ sequence was marked “▲”.

Phylogenetic analysis of Fads2 showed that the hybrid grouper clustered in the Fads2 group rather than Fads1 (Figure 5). Moreover, Fads2 in hybrid grouper shared high sequence identity with Fads2 of Perciformes, such as *Epinephelus coioides* (99%), *Epinephelus lanceolatus* (99%), *Lateolabrax japonicus* (88%), *Larimichthys crocea* (82%), and *Lepisosteus oculatus* (74%) and less similar to Fads1 such as *Anguilla japonica* (60%), *Polypterus senegalus* (59%) and *Lepisosteus oculatus* (56%).

### 3.3. Tissue Distribution of elovl5, elovl8 and fads2 mRNA Hybrid Grouper

**Figure 6:**
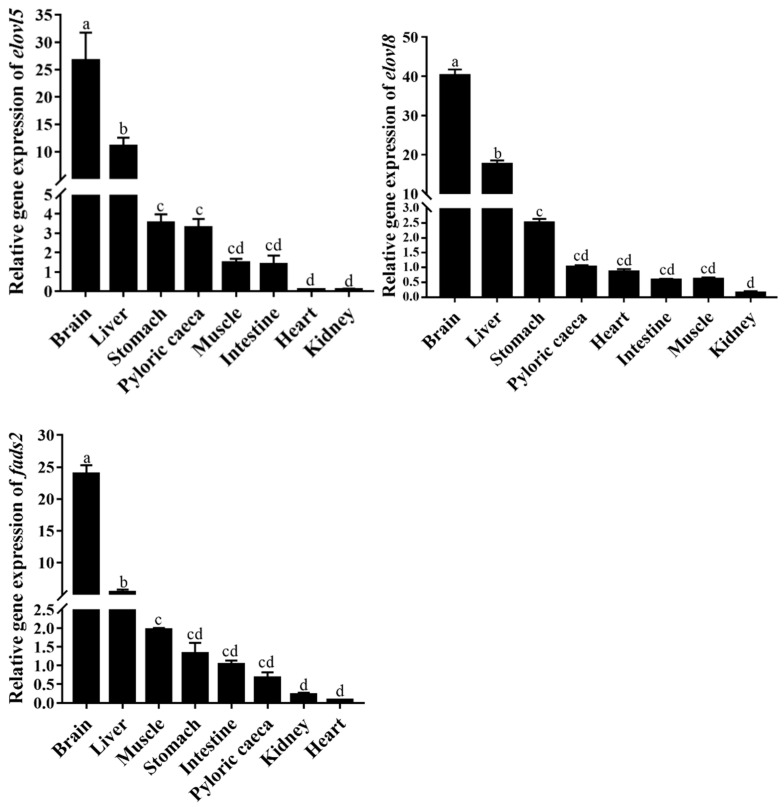
Tissue expression of *elovl5*, *elovl8* and *fads2* genes in hybrid grouper. Results are expressed as mean standard error (n = 4). Different letters above the bars denote significant (*p* < 0.05) differences among tissues.

As shown in Figure 6, the *elovl5*, *elovl8* and *fads2* genes were widely expressed in all the detected tissues of the hybrid grouper, including intestine, liver, muscle, brain, kidney, stomach, heart and pyloric caeca, with the highest expression in the brain (*p* < 0.05). Furthermore, moderate expression of *elovl5* and *elovl8* genes was found in the liver and stomach, while the *fads2* gene most highly expressed in liver and muscle. The results also showed that the heart and kidney were the tissues with the lowest gene expression of *elovl5* and *fads2* (*p* < 0.05), while the lowest value for *elovl8* gene expression was found in the kidney (*p* < 0.05).

### 3.4. Nutritional Regulation of Hybrid Grouper elovl5, elovl8 and fads2 Genes Expression

**Figure 7:**
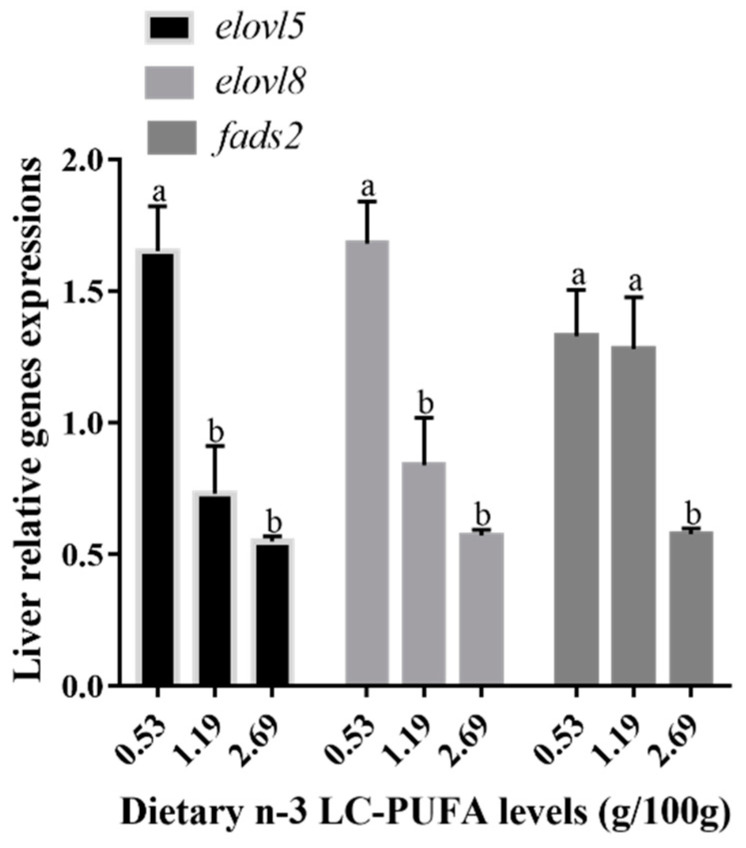
Relative mRNA expression of *elovl5*, *elovl8* and *fads2* in liver of hybrid grouper fed diets with different levels of n-3 LC-PUFA. Results are expressed as means standard error (n = 3). Different letters above the bars denote significant (*p* < 0.05) differences among dietary groups.

The gene expression of *elovl8*, *elovl5* and *fads2* in the liver of hybrid grouper were significantly affected by the dietary n-3 LC-PUFA levels (*p* < 0.05) (Figure 7). *Elovl5* and *elovl8* relative mRNA expressions were significantly decreased at 1.19 and 2.69 of dietary n-3 LC-PUFA levels compared with group 0.53 (*p* < 0.05). In contrast, a significantly downregulated gene expression of *fads2* was recorded in group 2.69 compared with other treatments (*p* < 0.05).

## 4. Discussion

Similar to other cultured marine fish species, fish oil is the primary lipid source for hybrid groupers. Considering the non-sustainable reliance on fish oil to farm this species, several trials have investigated the impact of the dietary replacement of fish oil with alternatives [30,31,32,33]. However, studies related to the molecular basis of LC-PUFA biosynthesis and regulation, which could optimize the pathway to the efficient use of alternatives, are still limited in hybrid groupers. Thus, we first cloned and characterized the main enzymes participating in the LC-PUFA biosynthesis in the present study, including Elovl5, Elovl8 and Fads2, from this fish species. To date, *elovl5* cDNA has been identified in numerous marine fish species, including Epinephelinae, and has been demonstrated to effectively elongate C18 and C20 PUFA [34]. In the present study, the isolated hybrid grouper *elovl5* cDNA sequence had 294 amino acids and showed high identity with other teleost *elovl5*, particularly Perciformes including *Epinephelus coioides* (99%), *Epinephelus lanceolatus* (99%), *Acanthopagrus schlegelii* (95%) and *Larimichthys crocea* (95%). The hybrid grouper Elovl5 possessed standard features for Elovl protein family members, including the so-called histidine box (HXXHH), the canonical C-terminal ER retention signal, several predicted transmembrane regions and other highly conserved motifs [35].

Elovl8a and elovl8b were first identified and functionally characterizated in rabbitfish from Li’s lab. Multiple sequences alignment showed that elovl8 shared a closer relationship with elovl4 [18]. Afterwards, Sun et al. conducted a phylogenetic analysis to establish the orthology of the newly identified *elovl8* gene and revealed that the similar orthologs from some fish species which were all annotated as *elovl4* or *elovl4*-like could be misidentified due to the sequence similarity to *elovl4* genes [9]. Thus, the previously similar *elovl* genes that have been annotated as “*elovl4*” (or “*elovl4*-like”) in many fish species were considered to be *elovl8* [9]. Similarly, amino acid sequence alignment with elovl4, elovl5 and elovl8 from other species showed that the new isolated “*elovl4*” gene from hybrid grouper was highly similar to those of elovl8b, and exhibited high identity with other teleost elovl8b, particularly Perciformes, including *Siganus canaliculatus* Elovl8b (86%), *Salmo salar* Elovl8b (83%), *Oncorhynchus mykiss* Elovl8b (83%), *Ictalurus punctatus* Elovl8b (81%) and *Danio rerio* Elovl8b (80%). Hybrid grouper Elovl8 possessed all the features of Elovl8 protein family members, including motifs (KXXEXXDT, QXXFLHXYHH, NXXXHXXMYXYY and TXXQXXQ), endoplasmic reticulum (ER) retention signal, multiple membrane-spanning regions and a histidine box (HXXHH), which is involved in the coordination of electron reception during fatty acid elongation [36]. It has been shown that the two Elovl8 isoforms may play various roles during the n-3 PUFA synthesis in different species. For instance, Elovl8b could elongate C18 (18:2n-6, 18:3n-3 and 18:4n-3) and C20 (20:4n-6 and 20:5n-3) polyunsaturated fatty acids (PUFAs) to longer-chain polyunsaturated fatty acids (LC-PUFAs), whereas Elovl8a lacked this ability in rabbitfish. However, in the study of zebrafish, Elovl8a activity was specific to C18-C20 PUFAs, just as Elovl4, Elovl5 and Elovl8b activity was specific to C18:0 and C20:1 MUFAs similar to Elovl1, Elovl3, and Elovl7 [9,19]. Thus, further studies need to explore Elovl8 functions in LC-PUFA synthesis in this hybrid grouper. 

Fads2 (Δ6 desaturase) catalyzes the first desaturation step in LC-PUFA synthesis and has been widely studied as the rate-limiting enzyme in the classical “Δ6 desaturation-Elovl5-Δ5 desaturation” LC-PUFA biosynthetic pathway [37]. Like all teleost fish species, phylogenetic analysis confirmed that the identified hybrid grouper cDNA was *fads2* rather than *fads1*, while both Fads1 and Fads2 desaturases were found in Elopomorpha species such as Japanese eel (*Anguilla japonica*), and it was considered that Fads1 was retained in Chondrichthyes and early ray-finned fish before its subsequent loss in Osteoglossomorpha and Clupeocephala [38]. As it is compensating for the lack of Fads1 in teleost genomes, Fads2 have functionally diversified during their evolution, such that Δ6, Δ4, Δ5, Δ8, or bifunctional desaturation abilities have been recorded in different fish species. Moreover, it has been demonstrated that the low expression of the *fads2* gene in carnivorous marine species was due to the lack of a binding site for stimulatory protein 1 (Sp1) [39]. 

The analysis of tissue distribution patterns for genes is helpful to better understand their physiological roles. Our study showed that the gene expression of *elovl5*, *elovl8*, and *fads2* was detected in all the examined tissues, which was consistent with the ubiquitous expression previously reported in fish [18,19,28]. Furthermore, these three genes were predominantly expressed in the brains of hybrid groupers, as reported in other carnivorous marine fish species, including groupers and other species, such as cobia [40], Asian seabass (*Lates calcarifer*) [41], Nibe croaker (*Nibea mitsukurii*) [22], and Northern pike (*Esox lucius*) [42]. However, inconsistently with the *elovl8b* distribution pattern in the tissues, the *elovl8a* was highly expressed in the heart and spleen of rabbitfish, which demonstrates their various roles in the LC-PUFA synthesis process. DHA is one of the most abundant LC-PUFA in the brain and is vital for brain function and development; this is the likely reason for *elovl5*, *elovl8* and *fads2* being highly expressed, to supply the high requirements for this tissue. However, studies in freshwater and marine herbivorous species such as rabbitfish and Atlantic salmon [11,18] showed that the liver and intestine, as the significant metabolic sites for LC-PUFA biosynthesis, exhibited the highest expression of *elovl5* and *fads2* genes. In addition, our study also showed that relatively higher gene expressions of *elovl5* were found in pyloric ceca compared with intestine and muscle, which indicated that it could be an important site of LC-PUFA, as reported in rainbow trout [43]. However, the gene expression in the kidney was the lowest among all the measured tissue, which was inconsistent with the orange-spotted grouper findings [44]. These results suggest that the diversification of fish LC-PUFA synthesis in different tissues could be associated with factors such as feeding habits, ecological habits, and species-specific evolutionary history [45].

Marine fish species’ requirement for LC-PUFA firstly depends on daily intake from the diet. As alternative oil sources lacking in LC-PUFA are gradually being used in the diet, it is essential to clarify the patterns of dietary fatty acids in regulating genes encoding desaturases and elongases in farmed fish. As the liver serves as the primary site for FA metabolism, including de novo synthesis of FA, we evaluated the gene expression of *elovl5*, *elovl8* and *fads2* in the liver of hybrid groupers, and they were down-regulated by higher dietary n-3 LC-PUFA levels, which was consistent with the findings in the orange-spotted grouper [28,29,44] and other fish species such as the large yellow croaker [46]. These observations reflect the negative feedback regulation of the LC-PUFA synthetic pathway. Atlantic salmon *elovl5* reporter activities were induced by the overexpression of LXRα, but not by the overexpression of sterol regulatory element-binding protein 1 (SREBP-1) [47]. In a study of Japanese seabass, CpG methylation of the *fads2* promoter was observed as hepatic *fads2* expression decreased in response to high dietary n-3 LC-PUFA, but a similar result was not recorded in European seabass [48]. Thus, further investigation is required to explore the regulation mechanism related to the different genes involved in LC-PUFA for hybrid groupers.

## 5. Conclusions

In summary, *elovl5*, *elovl8* and *fads2* genes were identified from hybrid groupers. The characteristics of the above genes were similar to other marine teleost fish species. Moreover, *elovl5*, *elovl8* and *fads2* were broadly expressed in most tissues, with the highest levels being found in the brain and liver, followed by the stomach, and the lowest levels being found in the kidney. Furthermore, the hepatic gene expression of *elovl5*, *elovl8* and *fads2* was downregulated by high dietary n-3 LC-PUFA. However, future studies are needed to determine the functional characterization of these genes and to explore the mechanism of these genes when regulated by the dietary fatty lipid profiles to better understand the process of n-3 LC-PUFAs biosynthesis in this fish species. 

## Figures and Tables

**Table 1:** Primers used for sequencing and qRT-PCR analysis of *elovl5*, *elovl8* and *fads2* from hybrid grouper.

Primer Name	Sequence (5′-3′)	Usage	TM	Expected Amplicon Size
Elovl5 F	ACCTTCTAATCGTGTGGATG	Partial sequence	51.9 °C	648 bp
Elovl5 R	TGTGCTTCTTGTAAGTCTGA	Partial sequence	51.3 °C	
Elovl5 5F	AAGCAGTGGTATCAACGCAGAGT	5’-RACE	59 °C	383 bp
Elovl5 5R	TCCACTTCCTGTGCACTGTGAGTGTC	5’-RACE	62.8 °C	
Elovl5 3F	CACACTCATCTTCCTCTTCTCAAACTT	3’-RACE	57.3 °C	466 bp
Elovl5 3R1	CTAATACGACTCACTATAGGGCAAGCAGTGGTATCAACGCAGAGT	3’-RACE	68 °C	
Elovl5 3R2	CTAATACGACTCACTATAGGGC	3’-RACE	51.8 °C	
Elovl8 F	ATGGCTGCTGGTCTACTC	Partial sequence	53.3 °C	677 bp
Elovl8 R	AGTTACTGAAGAGGATGATGAG	Partial sequence	51.4 °C	
Elovl8 5F	AAGCAGTGGTATCAACGCAGAGT	5’-RACE	59 °C	608 bp
Elovl8 5R	GGTGCCGTGGTGGTAAACATGAAGG	5’-RACE	62.5 °C	
Elovl8 3F	ATGCAGAAGTACCTGTGGTGGAAGAGA	3’-RACE	61.4 °C	538 bp
Elovl8 3R1	CTAATACGACTCACTATAGGGCAAGCAGTGGTATCAACGCAGAGT	3’-RACE	68 °C	
Elovl8 3R2	CTAATACGACTCACTATAGGGC	3’-RACE	51.8 °C	
Fads2 F	CACTACGCTGGAGAGGATG	Partial sequence	55.2 °C	608 bp
Fads2 R	TGGTGTTGGTGATGATAGG	Partial sequence	51.4 °C	
Fads2 5F	AAGCAGTGGTATCAACGCAGAGT	5’-RACE	59 °C	476 bp
Fads2 5R	CTCCGTGGCATCCTCTCCAGCGTAGT	5’-RACE	65.6 °C	
Fads2 3F	CATTTCCAGCATCACGCTAAACCCAAC	3’-RACE	60 °C	1117 bp
Fads2 3R1	CTAATACGACTCACTATAGGGCAAGCAGTGGTATCAACGCAGAGT	3’-RACE	68 °C	
Fads2 3R2	CTAATACGACTCACTATAGGGC	3’-RACE	51.8 °C	
elongation factor 1 alpha F	AGGGATGGAAGATTGAGCGC	Internal control	57.2 °C	74 bp
elongation factor 1 alphaR	CGTACCGGGCTTCAGGATAC	Internal control	57 °C	
Elovl8 RT-F	CAGATGATCCAGTTCCACGTCA	qRT-PCR	56.8 °C	525 bp
Elovl8 RT-R	GCGGTAGGTCTGGTAGTAGAAG	qRT-PCR	56.1 °C	
Elovl5 RT-F	CTACTGCCAGGACACTCACA	qRT-PCR	56 °C	415 bp
Elovl5 RT-R	GAGGCGCCAAAGTATGAGTG	qRT-PCR	56 °C	
Fads2 RT-F	CCAGGTGGAGGCAGAAGAACA	qRT-PCR	58.8 °C	184 bp
Fads2 RT-R	AGCCACTATGCTGGAGAGGATG	qRT-PCR	58.2 °C	
Elovl8 AF	AGCGTGCTCACTCACTTTTAACGG	ORF validation	57 °C	933 bp
Elovl8 AR	GTGTGCTTCTGCCTTCTCCATCCTT	ORF validation	59 °C	
Elovl5 AF	GTCGCTTTCTCTCCCCCGCCTCTCA	ORF validation	64 °C	953 bp
Elovl5 AR	CTACAGTGAGAATTGGGTGGCGGTTT	ORF validation	60 °C	
Fads2 AF	GCCAAAATCTGGATACTGTGTCAAA	ORF validation	54 °C	1628 bp
Fads2 AR	AAGACACTGTAAGGCAACCAGAGAAA	ORF validation	56 °C	

**Table 2:** Formulation and proximate analysis of the experimental diets.

Ingredients (g/100 g Diet)	Dietary n-3 PUFA Levels		
	0.53	1.19	2.69
Fish meal ^1^	22	22	22
Pork blood meal ^2^	6.0	6.0	6.0
Chicken meal ^3^	18	18	18
Casein ^4^	17.5	17.5	17.5
Palm oil ^5^	3.41	2.53	0.66
DHA purified oil ^6^	0.2	0.73	1.87
EPA purified oil ^7^	0	0.35	1.08
Vitamin premix ^8^	1	1	1
Mineral Premix ^9^	0.5	0.5	0.5
Starch	16	16	16
Cellulose	13.4	13.4	13.4
Crude protein	53.3	53.4	53.6
Crude lipid	7.12	6.93	7.1
n-3 PUFA	0.53	1.19	2.69

^1^ Yongsheng Feed Co., Ltd (Binzhou, Zhejiang province, China). Proximate composition (%Dry matter): Moisture, 7.43; Crude protein, 73.3; Crude lipid, 5.1; ^2^ Zhejiang Sonak Company (Chaozhou, Zhejiang province, China). Proximate composition (%Dry matter): Moisture, 6.46; Crude protein, 98.53; Crude lipid, 0.02; ^3^ Dalian Xin Ruisen Trading Company (Dalian, Liaoning province, China). Proximate composition (%Dry matter): Moisture, 4.38; Crude protein, 68.77; Crude lipid, 12.6; ^4^ Gansu Hualing Dairy Products Co., Ltd (Hezuo, Gansu province, China). Proximate composition (%Dry matter): Moisture, 4.38; Crude protein, 93.80; ^5^ Tianjin Julong Cereals and Oils Co., Ltd (Tianjin, China).; ^6^ Shaanxi Guanchen Biological Co., Ltd (Xian, Shaanxi province, China). Proximate composition (%Dry matter): Dh 75.1; EPA 4.8; ^7^ Shaanxi Guanchen Biological Co., Ltd (Xian, Shaanxi province, China). Proximate composition (%Dry matter): DHA 5.1; EPA 74.9; ^8^ Vitamin premix (mg/g premix): thiamin hydrochloride, 2.5; riboflavin, 10; calcium pantothenate, 25; nicotinic acid, 37.5; pyridoxine hydrochloride, 2.5; folic acid, 0.75; inositol, 100; ascorbic acid, 50; choline chloride, 250; menadione, 2; alpha-tocopheryl acetate, 20; retinol acetate, 1; cholecalciferol, 0.0025; biotin, 0.25; vitamin B12, 0.05. All ingredients were supplemented to 1 g with alpha cellulose; ^9^ Mineral Premix (mg/g mixture): calcium lactate, 327; K_2_PO_4_, 239.8; CaHPO_4_·2H_2_O, 135.8; MgSO_4_·7H_2_O, 132; Na_2_HPO_4_·2H_2_O, 87.2; NaCl, 43.5; ferric citrate, 29.7; ZnSO_4_·7H_2_O, 3; CoCl_2_·6H_2_O, 1; MnSO_4_·H_2_O, 0.8; KI, 0.15; AlCl_3_·6H_2_O, 0.15; CuCl_2_, 0.1; Note: The data represent the average of repeated samples.

**Table 3:** Fatty acid composition of experimental diets. The results are expressed as the average of repeated samples; SFA: saturated fatty acid; MUFA: monounsaturated fatty acid; PUFA: highly unsaturated fatty acid.

Fatty Acid	Dietary n-3 PUFA Levels		
	0.53	1.19	2.69
14:0	1.01	1.27	2.04
16:0	28.18	23.55	10.16
18:0	10.26	8.75	9.40
16:1n-9	0.97	2.12	3.18
18:1n-7	0.80	0.87	0.91
18:1n-9	20.75	17.33	19.48
20:1n-9	0.11	0.11	0.21
22:1n-9	0.11	0.10	0.10
18:2n-6 (LA)	18.13	13.06	5.99
18:3n-6	0.41	0.32	0.30
20:2n-6	0.10	0.20	0.31
20:4n-6 (ARA)	1.06	1.07	0.99
18:3n-3 (ALA)	1.07	1.00	1.01
20:5n-3 (EPA)	2.99	6.69	15.12
22:6n-3 (DHA)	4.61	10.28	23.29
∑SFA	39.45	33.57	21.60
∑MUFA	22.73	20.54	23.87
∑n-3 PUFA	7.60	16.97	38.41

## Data Availability

Data are available on request from the corresponding author.

## Supplementary Materials

The following are available online at https://www.mdpi.com/article/10.3390/ani12030234/s1. Figure S1: Multiple Sequence Alignment of *elovl5*, *elovl8* and *fads2* genes used for primer design. The design sequence of the primers is marked with square, Figure S2: Maximum likelihood method parameters, Figure S3: Effect of dietary n-3 LC-PUFA levels (g/100 g) on weight gain (%) of hybrid grouper, Figure S4: Nucleotide and deduced amino acid sequence of Elovl5 in hybrid grouper (GenBank accession number MZ713365). The nucleotide sequence is numbered from the first base at 5′ end, started in codon (ATG) and stopped in codon (TGA). Four conserved motif of elongases are in gray; the putative histidine-richdomain (HXXHH) is in bold; the predicted seven (I-VII) putative membrane-spanning domains are underlined; the ER retrieval signal is wavy underlined, Figure S5: Nucleotide and deduced amino acid sequence of Elovl8 in hybrid grouper (GenBank accession number MZ713364). The nucleotide sequence is numbered from the first base at 5′ end, started in codon (ATG) and stopped in codon (TGA). The putative transmembrane regions are in gray; the putative histidine-richdomain (HXXHH) is in bold; the predicted seven (I-VII) putative membrane-spanning domains are underlined; the ER retrieval signal is wavy underlined, Figure S6: Nucleotide and deduced amino acid sequence of Fads2 in hybrid grouper (GenBank accession number MZ713366). The nucleotide sequence is numbered from the first base at the 5′ end, started in codon (ATG) and stopped in codon (TGA). The Cytochrome b5-like Heme/Steroid binding domain is in gray; the putative histidine-rich domain (HXXHH, HDXGH and QXXHH) is in bold; the predicted four (I-IV) putative membrane-spanning domains are underlined.
