# Supplementary material for: Molecular Characterization, Tissue Distribution and Differential Nutritional Regulation of Three n-3 LC-PUFA Biosynthesis-Related Genes in Hybrid Grouper (Epinephelus fuscoguttatus ♀ × Epinephelus lanceolatus ♂)
Source: Animals (Basel). 2022 Jan 19;12(3):234. doi: 10.3390/ani12030234 (PMC8833367; doi:10.3390/ani12030234)
Supplement: Supplementary file 1 [file animals-12-00234-s001.zip › animals-1515421-supplementary.pdf]

```
Salmo salar elovl5  NM 001136552.1                 ACACTCATGTACTTGCTGATCGTATGGCTGGGGCCCAAGTACATGAGACACAGACAGCCAGTGTCTTGCCAGGGC
Epinephelus lanceolatus elovl5  XM 033613646.1     ACAGTCATGTACCTTCTAATCGTGTGGATGGGGCCCAAGTACATGAAACACAGACAGCCGTACTCCTGCAGAGGC
Epinephelus coioides elovl5  KU179484.1            ATTTCATGACCTTCAACAATTTAAAAGCCAGAGAATCACTACACAAACGTCTGGTTATTGTCCGGACGCACCTGC
Trachinotus ovatus elovl5  KY860144.1              ACAGTCATGTACCTTCTGATCGTGTGGATGGGGCCCAAGTACATGAAACACAGGCAGCCGTACTCCTGCAGAGGC
Rachycentron canadum elovl5  FJ440239.1            ACAGTAATGTACCTTCTGATCGTGTGGATGGGGCCCAAGTACATGAAACACAGGCAGCCCTATTCCTGCAGAGGC
                                                             ACCTTCTAATCGTGTGGATG

Salmo salar elovl5  NM 001136552.1                 CTCCTGGTGCTCTACAATCTGGCCCTCACTCTATTATCTTTCTATATGTTCTATGAGATGGTGTCTGCTGTGTGG
Epinephelus lanceolatus elovl5  XM 033613646.1     CTCCTGGTGCTTTACAATCTGGGCCTCACACTCTTGTCCTTCTACATGTTCTATGAGCTTGTTACCGCCGTGTGG
Epinephelus coioides elovl5  KU179484.1            CTGCTGGCTGCTTGTTTTACAGACACATTTGATCTGTTGTTTGAAACCACAGCGGTGTGGAAGATCAGTTATTGG
Trachinotus ovatus elovl5  KY860144.1              CTCCTGGTGCTCTACAATCTGGGCATCACACTCTTGTCTTTCTACATGTTCTATGAGCTTGTTTCTGCTGTGTGG
Rachycentron canadum elovl5  FJ440239.1            CTCCTGGTGCTCTACAATCTGGGCCTCACACTCTTGTCTTTCTACATGTTCTATGAGCTTGTTACTGCTGTGTGG
                                                                              TCCACTTCCTGTGCACTGTGAGTGTC

Salmo salar elovl5  NM 001136552.1                 CAAGGGGGTTATAACTTCTACTGCCAAGACACACACAGTGCGGAGAAACAGATACCAAGATCATAAATGTGCTG
Epinephelus lanceolatus elovl5  XM 033613646.1     CACGGTGGCTACAACTTCTACTGCCAGGACACTCACAGTGCACAGGAAGTGGATAATAAGATCATAAATGTCCTG
Epinephelus coioides elovl5  KU179484.1            GGTGTGAGATGTATTAGAGAGTGTGAGTGGAGAGGCTGGAGCACAGACATTTACAACAGACACAATAGGGCTGGC
Trachinotus ovatus elovl5  KY860144.1              CACGGTGGTTACAACTTTTACTGCCAGGACACTCACAGTGCACAGGAAGTGGATAATAAGATTATAAATGTCCTG
Rachycentron canadum elovl5  FJ440239.1            CATGGTGGCTACAACTTTTACTGCCAGGACACTCACAGTGCAGAGGAAGTGGATAATAAGATTATAAATGTCCTG
                                                                  CTACTGCCAGGACACTCACA

Salmo salar elovl5  NM 001136552.1                 TGGTGGTACTACTTCTCCAAGGTCATAGAGTTTATGGACACCTTCTTCTTCATCCTACGGAAGAACAACCATCAG
Epinephelus lanceolatus elovl5  XM 033613646.1     TGGTGGTACTACTTCTCCAAGCTCATCGAGTTCATGGACACCTTCTTCTTCATACTACGAAAGAATAACCACCAG
Epinephelus coioides elovl5  KU179484.1            CCGGTATATCCATATTATATCTAAATTGTGATTGTGT. TGTCTTAGACTTTGGATATTGTAAAATCGTACATATG
Trachinotus ovatus elovl5  KY860144.1              TGGTGGTACTACTTCTCAAAGCTCATTGAGTTCATGGACACATTTTTCTTCATACTACGGAAGAATAACCATCAG
Rachycentron canadum elovl5  FJ440239.1            TGGTGGTACTACTTCTCCAAGCTGATTGAGTTCATGGACACATTTTTCTTCATACTACGAAAGAATAATCACCAG

Salmo salar elovl5  NM 001136552.1                 ATCACGTTTCTGCACATCTACCACCACGCTAGCATGCTCAACATCTGGTGGTTCGTCATGAACTGGGTGCCCTGT
Epinephelus lanceolatus elovl5  XM 033613646.1     ATCACGTTTCTTCACATCTACCACCACGCTAGCATGCTGAATATCTGGTGGTTCGTTATGAACTGGGTACCCTGC
Epinephelus coioides elovl5  KU179484.1            GTCTTGT. . CCTGATTTTAAAGGCTGCATTA. CAGCAAGGTGATATAATTTTCTGAACTGACCAGACTGTTCTCG
Trachinotus ovatus elovl5  KY860144.1              ATCACATTTCTTCACATCTACCACCACGCTACCATGCTGAATATCTGGTGGTTTGTTATGAACTGGATACCCTGT
Rachycentron canadum elovl5  FJ440239.1            ATCACATTTCTTCACATCTACCACCATGCCACCATGCTGAATATATGGTGGTTTGTTATGAACTGGATACCCTGT

Salmo salar elovl5  NM 001136552.1                 GGTCACTCCTACTT. . TGGTGCCTCCCTCAACAGCTTCGTCCATGTCCTGATG. TACTCTTACTATGGGCTCTCT
Epinephelus lanceolatus elovl5  XM 033613646.1     GGCCACTCATACTT. . TGGCGCCTCCCTAAACAGCTTCGTCCACGTCGTTATG. TATTCTTACTACGGCCTGTCA
Epinephelus coioides elovl5  KU179484.1            CATTTTTATTATCTGACTTTACCTACTTAGTCATTATATTCCCATTACTGATGATATTATCAATATTAAG. GTAT
Trachinotus ovatus elovl5  KY860144.1              GGCCATTCGTACTT. . TGGTGCATCCCTGAACAGCTTTGTCCACGTTGTGATG. TATTCCTACTACGGCCTCTCA
Rachycentron canadum elovl5  FJ440239.1            GGCCATTCGTACTT. . CGGTGCATCGCTAAACAGCTTCGTCCACGTTGTGATG. TATTCTTACTACGGCCTCTCA
                                                     GAGGCGCCAAAGTATGAGTG

Salmo salar elovl5  NM 001136552.1                 GCTGTCCCGGCCATACGGCCCTATCTATGGTGGAAGAA. . . ATATATCACACAAGGCCAGCTGATTCAATTCTTT
Epinephelus lanceolatus elovl5  XM 033613646.1     GCCATCCCAGCCATCCGGCCGTACCTTTGGTGGAAGAA. . . GTACATCACACAGTTTCAGCTGATCCAGTTCTTT
Epinephelus coioides elovl5  KU179484.1            ATTGTCAAAAATATTTCATTTTTTCCATATTGTCCAGCCCTAGACATGATGGATATGTATCAAAAAGGACAAGTT
Trachinotus ovatus elovl5  KY860144.1              TCCATCCCAGCCGTGCGGCCATACCTTTGGTGGAAGAA. . . ATACATCACGCAGTTACAGCTGATCCAGTTCTTT
Rachycentron canadum elovl5  FJ440239.1            GCCATTCCAGCCATGCGGCCGTACCTTTGGTGGAAGAA. . . ATACATCACACAGTTACAGCTGATCCAGTTCTTC

Salmo salar elovl5  NM 001136552.1                 TTGACCATGTCCCAGACCATCTGTGCAGTCATTTGGCCATGTGGTTTCCCCAGAGGCTGGCTGTTTTTCCAAATA
Epinephelus lanceolatus elovl5  XM 033613646.1     TTAACCATGTCGCAGACGATGTGCGCGGTCATATGGCCATGCGGCTTCCCCAAGGGATGGCTGTACTTCCAAATA
Epinephelus coioides elovl5  KU179484.1            TTATTTATGTATGTATTTTTTAATACTGCATAGAGTTAATCAGAATTTACAGAAGAATGTTGTCATGATCACTTC
Trachinotus ovatus elovl5  KY860144.1              TTAACCATGTCACAGACAATGTGTGCAGTCATATGGCCATGTGGCTTCCCCATGGGATGGCTGTACTTCCAAATA
Rachycentron canadum elovl5  FJ440239.1            TTGACCATGTCCCAGACAATGTGTGCTGTCATATGGCCATGTGATTTCCCCAGAGGATGGCTATACTTTCAAATA

Salmo salar elovl5  NM 001136552.1                 TTCTATATGGCCTCGCTTATTGCCTTTTTCTCAAACTTCTACATTCAGACTTACAAGAAACACCGTGTTTCACAA
Epinephelus lanceolatus elovl5  XM 033613646.1     AGTTACATGGTCACACTCATCTTCCTCTTCTCAAACTTCTACGTTCAGACTTACAAGAAGCACAGTGGCTCTCTA
Epinephelus coioides elovl5  KU179484.1            GCCCTTGAGCCCTCATTTAGCCAGATTAATTAAGATTATCATACTTGAAGCTGCACTAATCAATACTTTTCTACA
Trachinotus ovatus elovl5  KY860144.1              AGTTACATGGTCACGCTCATTATCCTTTTCTCAAACTTCTACATTCAGACGTACAAGAAGCAGAGTGGTTCCCTA
Rachycentron canadum elovl5  FJ440239.1            AGTTACATGGTCACACTCATTATCCTTTTCTCAAACTTCTACATTCAGACGTACAAGAAACACAGTGGTTCTCTA
                                                             CACACTCATCTTCCTCTTCTCAAACTT  TGTGCTTCTTGTAAGTCTGA

Epinephelus lanceolatus elovl4-like  XM 033621466.1  GTGTCCTGTCTGTGCACCAGAGGATAGTGAACAATGGAGACAAGAGGACGGACCCATGGCTGCTGGTCTACTCCC
Trachinotus ovatus elovl4-like  MK215859.1           GTGTCCTGTCTGTGCACCAGAGGATAGTGGACAATGGAGACAAGAGGACTGACCCGTGGCTGCTGGTCTACTCCC
Salmo salar elovl4-like  XM 014215900.1              GCGTTCAGTCCATGCGCCAGTGGATTTAGAGAATGGAGATAAAAGGACAGACCCATGGCTACTGGTCTACTCCC
                                                                                                         ATGGCTGCTGGTCTACTC

Epinephelus lanceolatus elovl4-like  XM 033621466.1  CCGTCCCGGTGGCACTCATCTTCCTAGTGTACCTCTGTGTGGTCTGGGCCGGCCCTCGTCTGATGAAACACAGAG
Trachinotus ovatus elovl4-like  MK215859.1           CCGTCCCCGTGTCACTCATCTTTCTGGTTTACCTCTGTGTGGTCTGGGCCGGCCCTCGCCTGATGAAACGCAGGG
Salmo salar elovl4-like  XM 014215900.1              CCATGCCAGTGGCCTTTATATTCCTCCTCTATCTTGGTGTGGTCTGGGCTGGGCCCAAGCTGATGAAACGCAGGG
                                           CAGATGATCCAGTTCCACGTCA

Epinephelus lanceolatus elovl4-like  XM 033621466.1  AACCCGTTGACCTCAAAGTGGTTCTCATTGTTTACAACTTTGCCATGGTCGGCCTGTCTGCCTATATGTGCTATG
Trachinotus ovatus elovl4-like  MK215859.1           AACCAGTTGACCTCAAAGTCGTTCTCATTGTTTACAACTTCGCCATGGTCGGCCTGTCTGCCCACATGTTCTATG
Salmo salar elovl4-like  XM 014215900.1              AACCAGTTGATCTCAAGGCTGTACTCATTGTCTACAACTTCGCCATGGTCTGCCTGTCTGTCTACATGTTCCACG

Epinephelus lanceolatus elovl4-like  XM 033621466.1  AGTTCCTGGTCACATCCTGGCTCTCGAGCTACAGCCTCCTCTGTCAGCCTGTAGATTACAGCACCAACCCGCTAG
Trachinotus ovatus elovl4-like  MK215859.1           AGTTCCTGGTCACTTCATGGCTCTCAAACTACAGCTACCTCTGTCAGCCTGTAGATTACAGCACCAGCCCACTGG
Salmo salar elovl4-like  XM 014215900.1              AGTTCTTGGTCACGTCCTTGCTGTCTAACTACAGTTACCTGTGTCAACCTGTGGATTACAGCACTAGTCCACTGG

Epinephelus lanceolatus elovl4-like  XM 033621466.1  CATTGAGGATGGCCAGAGTCTGCTGGTGGTTCTTCTTCTCTAAGGTCATAGAGCTCAGTGACACCATCTTCTTCA
Trachinotus ovatus elovl4-like  MK215859.1           CGATGAGGATGGCCAGAGTCTGCTGGTGGTTTTTCTTCTCCAAGGTGATCGAGCTCTCTGACACGCTCTTCTTCA
Salmo salar elovl4-like  XM 014215900.1              CGATGAGGATGGCCAAAGTATGCTGGTGGTTTTTCTTCTCCAAGGTCATAGAATTGGCTGACACGGTGTTCTTCA

Epinephelus lanceolatus elovl4-like  XM 033621466.1  TTCTGAGGAAGAAGAACAGCCAGCTGACGTTCCTTCATGTTTACCACCACGGCACCATGATCTTCAACTGGTGGG
Trachinotus ovatus elovl4-like  MK215859.1           TCCTGAGGAAGAAGAACAGTCAGCTGACTTTCCTTCATGTCTACCACCACGGCACCATGATCTTCAACTGGTGGG
Salmo salar elovl4-like  XM 014215900.1              TCCTGAGGAAGAAGAACAGTCAGCTGACTTTCCTGCATGTCTATCACCATGGCACCATGATCTTCAACTGGTGGG
                                                                                   GGTGCCGTGGTGGTAAACATGAAGG

Epinephelus lanceolatus elovl4-like  XM 033621466.1  CGGGAGTCAAGTACGTGGCCGGCGGACAGTCGTTCTTCATCGGCCTGGTCAACACCTTAGTTCACATCGTGATGT
Trachinotus ovatus elovl4-like  MK215859.1           CGGGAGTCAAGTACGTGGCTGGAGGACAGTCGTTCTTCATTGGCCTGGTCAACACCTTTGTTCACATAGTGATGT
Salmo salar elovl4-like  XM 014215900.1              CAGGGGTCAAGTATCTGGCTGGAGGCCAATCGTTCTTCATCGGCCTGCTCAATACCTTTGTGCACATCGTGATGT

Epinephelus lanceolatus elovl4-like  XM 033621466.1  ATTCTTACTACGGCCTGGCTGCGCTCGGCCCTCACATGCAGAAGTACCTGTGGTGGAAGAGATACCTCACCTCTC
Trachinotus ovatus elovl4-like  MK215859.1           ACTCTTACTACGGCCTGGCAGCGATCGGCCCTCACATGCAGAAGTACCTGTGGTGGAAACGATACCTCACGTCTC
Salmo salar elovl4-like  XM 014215900.1              ACTCTTACTACGGACTGGCTGCCCTGGGGCCTCACACGCAGAAGTACTTATGGTGGAAGCGCTATCTGACCTCGC
                                                                                       ATGCAGAAGTACCTGTGGTGGAAGAGA

Epinephelus lanceolatus elovl4-like  XM 033621466.1  TGCAGCTGGTGCAGTTTGTGCTGTTCCTCCTGCACACGGGCTACAACCTGTTCACAGAGTGCGACTTCCCCGACT
Trachinotus ovatus elovl4-like  MK215859.1           TGCAGCTGCTGCAGTTTCTGATGTTCCTTGTGCACACGGGCTACAACCTGTTCACAGAGTGTGACTTCCCCGACA
Salmo salar elovl4-like  XM 014215900.1              TGCAGCTGCTCCAGTTTGTCCTGTTGACCACTCACACTGGCTACAACCTCTTCACTGAGTGTGACTTCCCAGACT
                                                                                                                GCGGTAGGTCTGGTAGTAGAAG

Epinephelus lanceolatus elovl4-like  XM 033621466.1  CCATGAACGTGGTCGTGTTTGGTTACTGCGTCACCCTCATCATCCTCTTCAGTAACTTCTATTTTCAGAGCTACC
Trachinotus ovatus elovl4-like  MK215859.1           CCATGAACATGGTGGTGTTTGGTTACTGCGTCACCCTCATCATCCTCTTCAGTAACTTCTATTATCAGAGCTACC
Salmo salar elovl4-like  XM 014215900.1              CCATGAATGCTGTGGTGTTTGCCTACTGTGTCAGTCTCATTGCTCTCTTCAGCAACTTCTACTATCAGAGCTACC
                                                                                         AGTTACTGAAGAGGATGATGAG

Epinephelus lanceolatus elovl4-like  XM 033621466.1  TCAACAAGAAGA. . . AGCAGAAATAAGACACA. . . GTGATTGTGGAGCTTTA. . . . . . . . . TGAAACTGAAC. AG
Trachinotus ovatus elovl4-like  MK215859.1           TCAGCAAGAAGA. . . AGCAGAAATAAGATGCA. . . ATGGTGAGGCGTTGACA. . . . . . . . . CACAATCGAAC. AC
Salmo salar elovl4-like  XM 014215900.1              TCAACAGGAAGAGTAAGAAGACATAAAGGATATGGACAATCATGCACTCACAGTGTCTTGCTGGACCTGAATTAA
```

```
Trachinotus ovatus fads2  MG674449.1              ATGGGAGGTGGAGGTCAGCTGACGGAG. . . . . . . . . . . . . . . . . GGGCGAGCTGGAGGTGTCTACACCTGGGA
Epinephelus coioides fads2  EU715405.1           GATGGGAGGTGGAGGCCAGCTGACGGAGCCAGGGGAGCAGGGCAGTGGGCGGGCTGGTGGTGTCTACACCTGGGA
Epinephelus lanceolatus fads2  XM 033635131.1    GATGGGAGGTGGAGGCCAGCTGACGGAGCCAGGGGAGCAGGGCAGTGGGCGGGCTGGTGGTGTCTACACCTGGGA
                                                          CCAGGTGGAGGCAGAAGAACA

Trachinotus ovatus fads2  MG674449.1             GGAGGTGCAGAGTCACAATATCAGGAACGACCAGTGGCTGGTCATAGACCGAAAGGTTTACAACATCACTCAGTG
Epinephelus coioides fads2  EU715405.1           GGAGGTGCAGAGTCACTGCGACAGGAATGACCAGTGGCTGGTGATCGATCGGAAGGTTTACAACATCACACAGTG
Epinephelus lanceolatus fads2  XM 033635131.1    GGAGGTGCAGAGTCACTGCGACAGGAATGACCAGTGGCTGGTGATCGATCGGAAGGTTTACAACATCACACAGTG
                                                                        CACTACGCTGGAGAGGATG

Trachinotus ovatus fads2  MG674449.1             GGCCAAAAGGCATCCAGGAGGGTTTCGTGTCATCAGCCACTATGCTGGAGAGGACGCCACAGAGGCTTTCACTGC
Epinephelus coioides fads2  EU715405.1           GGCTAAAAGGCACCCAGGAGGGATCCGGGTCATCAGCCACTATGCTGGAGAGGATGCCACGGAGGCGTTCACCGC
Epinephelus lanceolatus fads2  XM 033635131.1    GGCTAAAAGGCACCCAGGAGGGATCCGGGTCATCAGCCACTATGCTGGAGAGGATGCCACGGAGGCGTTCACCGC
                                                       AGCCACTATGCTGGAGAGGATG  CTCCGTGGCATCCTCTCCAGCGTAGT

Trachinotus ovatus fads2  MG674449.1             TTTTCATCCTGATCCAACTTTTGTACAAAAGTTTCTGAAGCCCCTGCAGATTGGTGAGCTGGCAGCGACAGAGCC
Epinephelus coioides fads2  EU715405.1           TTTTCATCCTGATTTAAAGTTTGTGCAAAAGTTTCTGAAGCCCCTGCTGATCGGAGAGCTGGCAGCGACAGAGCC
Epinephelus lanceolatus fads2  XM 033635131.1    TTTTCATCCTGATTTAAAGTTTGTGCAAAAGTTTCTGAAGCCCCTGCTGATCGGAGAGCTGGCAGCGACAGAGCC

Trachinotus ovatus fads2  MG674449.1             CAGCCAGGACCGGAACAAAAATGCAGCAATCATACAGGATTTCCACACTTTACGTGCACAGGCAGAGAGCGAGGG
Epinephelus coioides fads2  EU715405.1           CAGCCAGGACCGAGACAAAAACGCAACAATCATACAAGATTTCCACACTTTACGGGTGCAGGCGGAGAGGGAGGG
Epinephelus lanceolatus fads2  XM 033635131.1    CAGCCAGGACCGAGACAAAAACGCAACAATCATACAAGATTTCCACACTTTACGGGTGCAGGCGGAGAGGGAGGG

Trachinotus ovatus fads2  MG674449.1             TCTGTTTCGAGCTCGGCCTTTGTTCTTCTGCCTCCACCTGGGTCACATCATGCTGCTGGAGGCCTTCGCCTGGCT
Epinephelus coioides fads2  EU715405.1           TTTGTTTCAAGCTCGGCCTTTGTTCTTCTGCCTCCACCTGGGTCACATCCTGCTGCTGGAGGCCCTCGCCTGGCT
Epinephelus lanceolatus fads2  XM 033635131.1    TTTGTTTCAAGCTCGGCCTTTGTTCTTCTGCCTCCACCTGGGTCACATCCTGCTGCTGGAGGCCCTCGCCTGGCT

Trachinotus ovatus fads2  MG674449.1             GATGATATGGCACTGGGGAACTAACTGGATACTGACGCTTCTGTGTGCGGTCATGCTGGCAACCGCTCAG. . . CA
Epinephelus coioides fads2  EU715405.1           CATCATTTGGGTCTGGGGAACCAGCTGGACGCTGACGTTTCTGATCTCCGTCATGCTGGCGACCGCTCAGTTGCA
Epinephelus lanceolatus fads2  XM 033635131.1    CATCATCTGGGTCTGGGGAACCAGCTGGACGCTGACGTTTCTGATCTCCTTCATGCTGGCGACCGCTCAGTTGCA

Trachinotus ovatus fads2  MG674449.1             GGCTGGTTGGCTGCAGCACGACTTCGGCCACCTGTCTGTCTTCAAGAAGTCCCGCTTGAATCACTTGTTGCACAA
Epinephelus coioides fads2  EU715405.1           GGCTGGATGGCTGCAGCACGACTTTGGCCACCTGTCTGTCTTCAAGAAGTCCAGCTGGAATCACATATTGCACAA
Epinephelus lanceolatus fads2  XM 033635131.1    GGCTGGATGGCTGCAGCACGACTTTGGCCACCTGTCTGTCTTCAAGAAGTCCAGCTGGAATCACATATTGCACAA

Trachinotus ovatus fads2  MG674449.1             GTTCACCATCGGCCATTTAAAGGGAGCTTCTGCCAACTGGTGGAATCATCGACATTTCCAGCATCATGCTAAACC
Epinephelus coioides fads2  EU715405.1           GTTTGCCATTGGCCATTTGAAGGGAGCGTCTGCCAACTGGTGGAATCACCGGCATTTCCAGCATCACGCTAAACC
Epinephelus lanceolatus fads2  XM 033635131.1    GTTTGCCATCGGCCATTTGAAGGGAGCATCTGCCAACTGGTGGAATCACCGGCATTTCCAGCATCACGCTAAACC
                                                                                              CATTTCCAGCATCACGCTAAACCCAAC

Trachinotus ovatus fads2  MG674449.1             CAATGTCTTCAGGAAGGACCCTGATGTCAACATGTTGAGTATTTTTGTTGTTGGATCCACTCAACCTGTGGAGTA
Epinephelus coioides fads2  EU715405.1           CAACATCTTCAGTAAGGACCCTGATGTCAACATGTTGCACATCTTTGTAGTTGGAACCACTCAACCAGTGGAGTA
Epinephelus lanceolatus fads2  XM 033635131.1    CAACATCTTCAGTAAGGACCCTGATGTCAACATGTTGCACATCTTTGTAGTTGGAACCACTCAACCAGTGGAGTA

Trachinotus ovatus fads2  MG674449.1             TGGCATAAAAAAGATCAAACACATGCCCTACCATCACCAACACCAGTACTTCTTTCTTGTGGACCACCGCTACT
Epinephelus coioides fads2  EU715405.1           CGGCATAAAGAAGATTAAATATATGCCCTATCATCACCAACACCAGTACTTCTTTCTTGTTGGACCACCGCTGCT
Epinephelus lanceolatus fads2  XM 033635131.1    CGGCATAAAGAAGATTAAATATATGCCCTATCATCACCAACACCAGTACTTCTTTCTTGTTGGACCACCGCTGCT
                                                                    TGGTGTTGGTGATGATAGG
```

Figure S1. Multiple Sequence Alignment of *elovl5, elovl8* and *fads2* genes used for primer design. The design sequence of the primers is marked with square.

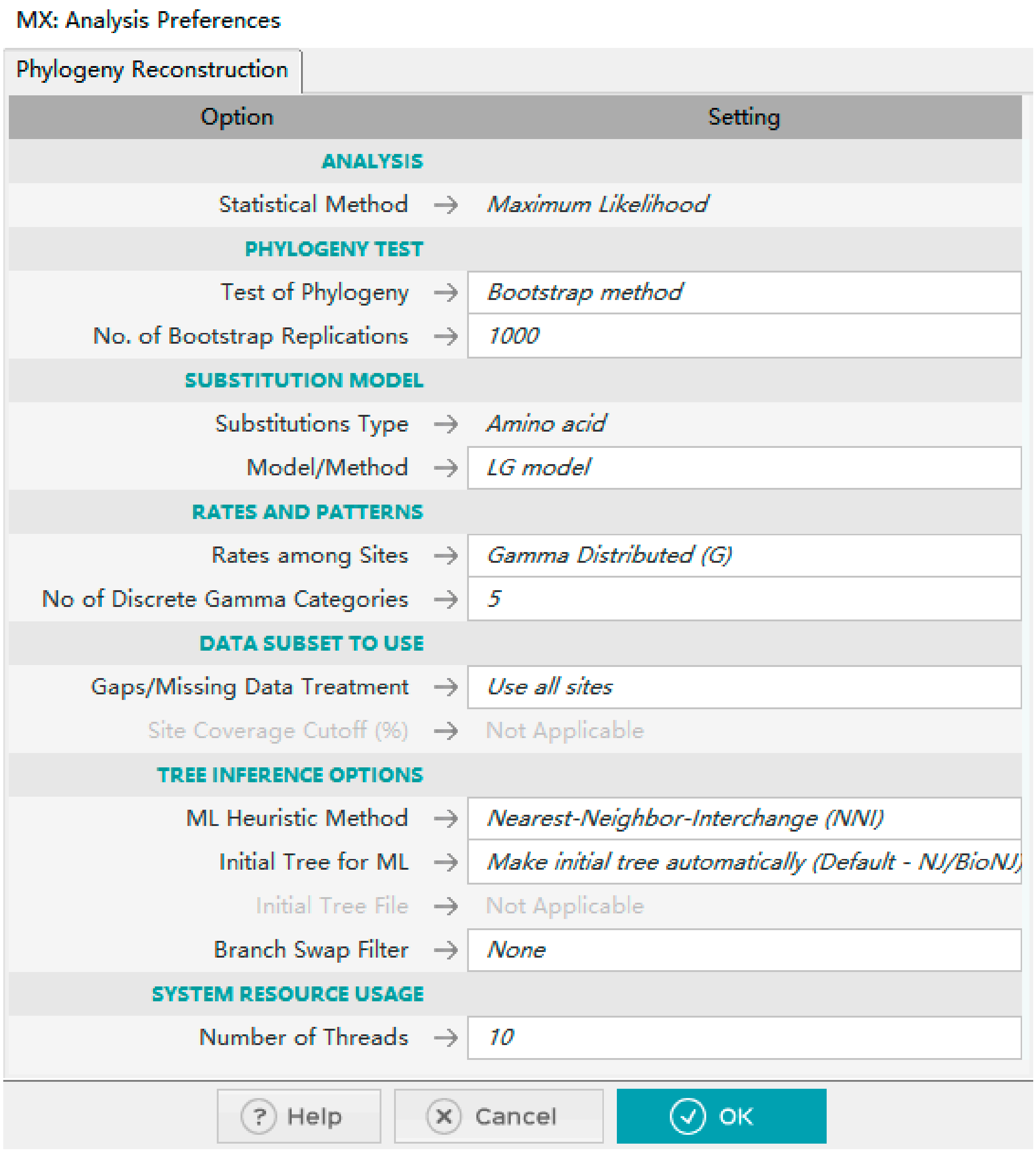

Figure S2. Maximum likelihood method parameters.

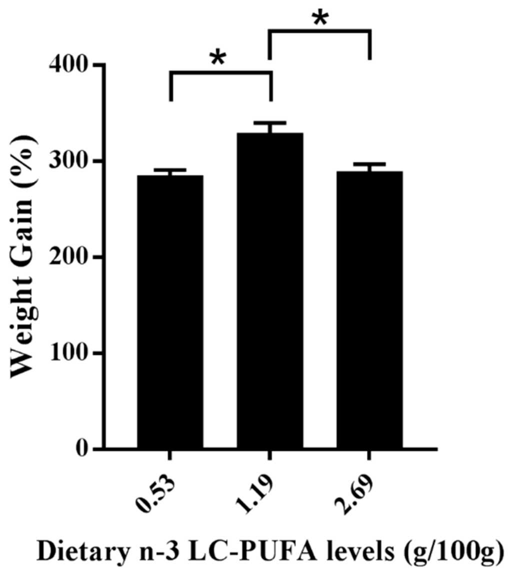

Figure S3. Effect of dietary n-3 LC-PUFA levels (g/100g) on weight gain (%) of hybrid grouper

```
ATGGGGAGTCGCTTTCTCTCCCCCGCCTCTCAAGGTGACAAATGGAGACCTTCAATCATAAACTAAACACATACTTAGAGTCATGGATGG 90
                                             M  E  T  F  N  H  K  L  N  T  Y  L  E  S  W  M  16
GCCCCAGGGATCAGAGGGTGCGGGGATGGCTGCTGCTCGACAACTACCCACCAACCTTTGCACTCACAGTCATGTACCTTCTAATCGTGT 180
G  P  R  D  Q  R  V  R  G  W  L  L  L  D  N  Y  P  P  T  F  A  L  T  V  M  Y  L  L  I  V  46
GGATGGGGCCCAAGTACATGAAACACAGACAGCCGTACTCCTGCAGAGGCCTCCTGGTGCTTTACAATCTGGGCCTCACACTCTTGTCCT 270
W  M  G  P  K  Y  M  K  H  R  Q  P  Y  S  C  R  G  L  L  V  L  Y  N  L  G  L  T  L  L  S  76
TCTACATGTTCTATGAGCTTGTTACCGCCGTGTGGCACGGTGGCTACAACTTCTACTGCCAGGACACTCACAGTGCACAGGAAGTGGATA 360
F  Y  M  F  Y  E  L  V  T  A  V  W  H  G  G  Y  N  F  Y  C  Q  D  T  H  S  A  Q  E  V  D  106
ATAAGATCATAAATGTCCTGTGGTGGTACTACTTCTCCAAGCTCATCGAGTTCATGGACACCTTCTTCTTCATACTACGAAAGAATAACC 450
N  K  I  I  N  V  L  W  W  Y  Y  F  S  K  L  I  E  F  M  D  T  F  F  F  I  L  R  K  N  N  136
ACCAGATCACGTTTCTTCACATCTACCACCACGCTAGCATGCTGAATATCTGGTGGTTCGTTATGAACTGGGTACCCTGCGGCCACTCAT 540
H  Q  I  T  F  L  H  I  Y  H  H  A  S  M  L  N  I  W  W  F  V  M  N  W  V  P  C  G  H  S  166
ACTTTGGCGCCTCCCTAAACAGCTTCGTCCACGTCGTTATGTATTCTTACTACGGCCTGTCAGCCATCCCAGCCATCCGGCCGTACCTTT 630
Y  F  G  A  S  L  N  S  F  V  H  V  V  M  Y  S  Y  Y  G  L  S  A  I  P  A  I  R  P  Y  L  196
GGTGGAAGAAGTACATCACACAGTTTCAGCTGATCCAGTTCTTTTTAACCATGTCGCAGACGATGTGCGCGGTCATATGGCCATGCGGCT 720
W  W  K  K  Y  I  T  Q  F  Q  L  I  Q  F  F  L  T  M  S  Q  T  M  C  A  V  I  W  P  C  G  226
TCCCCAAGGGATGGCTGTACTTCCAAATAAGTTACATGGTCACACTCATCTTCCTCTTCTCAAACTTCTACGTTCAGACTTACAAGAAGC 810
F  P  K  G  W  L  Y  F  Q  I  S  Y  M  V  T  L  I  F  L  F  S  N  F  Y  V  Q  T  Y  K  K  256
ACAGTGGCTCTCTAAAGAAGGAGCACCAAAACGGCTCTCCTGCATCTACAAATGGACATGCAAATGGGACGCCGTCAATGGAGCACACCG 900
H  S  G  S  L  K  K  E  H  Q  N  G  S  P  A  S  T  N  G  H  A  N  G  T  P  S  M  E  H  T  286
CACACAAGAAGCTGAGGGTGGATTGACATTTGAGAAACCGCCACCCAATTCTCACTGTAGCGTGTTAGCTAATGCCGCTAGGAGGTTTAA 990
A  H  K  K  L  R  V  D  -                                                                294
GTATCTTCTTATCTAGAATAGTTTAGCGCTCACATGAGATGAAATAAGCCATAGCCACATATATCCAGAGACTTTCCATGTTTTTGCACA 1080
CGTTCCTACTCATGGTATTTAATTATTAAATGAATATTAGTATATAAGGAGAGTATTGTAGTATGGTTGCATAATATTGCCTCCCCCAAC 1170
CCTCTAGAGGAAATTCACTCCAAAAAAAAAAAAAAAAAAAAAAAAAAAAAAAAAAA                                   1226
```

Figure S4. Nucleotide and deduced amino acid sequence of Elovl5 in hybrid grouper (GenBank accession number MZ713365). The nucleotide sequence is numbered from the first base at 5′ end, started in codon (ATG) and stopped in codon (TGA). Four conserved motif of elongases are in gray; the putative histidine-richdomain (HXXHH) is in bold; the predicted seven (I-VII) putative membrane-spanning domains are underlined; the ER retrieval signal is wavy underlined.

```
ATGGGGGCTGATACTGGTTTGCCAGGTGGCGTTCAAAGGTTCTCCAGCGTGCAGCGTGCTCACTCACTTTTAACGGTAAAACACCTGCTG 90
CAAGGTGTGCATCATTTTCAGATGGCTTTTGCATGGCAAAGTGTCCTGTCTGTGCACCAGAGGATAGTGGACAATGGAGACAAGAGGACG 180
                           M  A  F  A  W  Q  S  V  L  S  V  H  Q  R  I  V  D  N  G  D  K  R  T  23
GACCCATGGCTGCTGGTCTACTCCCCCGTCCCGGTGGCACTCATCTTCCTAGTGTACCTCTGTGTGGTCTGGGCCGGCCCTCGTCTGATG 270
D  P  W  L  L  V  Y  S  P  V  P  V  A  L  I  F  L  V  Y  L  C  V  V  W  A  G  P  R  L  M  53
AAACACAGAGAACCCGTTGACCTCAAAGTGGTTCTCATTGTTTACAACTTTGCCATGGTCGGCCTGTCTGCCTATATGTGCTATGAGTTC 360
K  H  R  E  P  V  D  L  K  V  V  L  I  V  Y  N  F  A  M  V  G  L  S  A  Y  M  C  Y  E  F  83
CTGGTCACATCCTGGCTCTCGAGCTACAGCCTCCTCTGTCAGCCTGTAGATTACAGCACCAACCCGCTAGCATTGAGGATGGCCAGAGTC 450
L  V  T  S  W  L  S  S  Y  S  L  L  C  Q  P  V  D  Y  S  T  N  P  L  A  L  R  M  A  R  V  113
TGCTGGTGGTTCTTCTTCTCTAAGGTCATAGAGCTCAGTGACACCATCTTCTTCATTCTGAGGAAGAAGAACAGCCAGCTGACGTTCCTT 540
C  W  W  F  F  F  S  K  V  I  E  L  S  D  T  I  F  F  I  L  R  K  K  N  S  Q  L  T  F  L  143
CATGTTTACCACCACGGCACCATGATCTTCAACTGGTGGGCGGGAGTCAAGTACGTGGCCGGCGGACAGTCGTTCTTCATCGGCCTGGTC 630
H  V  Y  H  H  G  T  M  I  F  N  W  W  A  G  V  K  Y  V  A  G  G  Q  S  F  F  I  G  L  V  173
AACACCTTTGTTCACATCGTGATGTATTCTTACTACGGCCTGGCTGCGCTCGGCCCTCACATGCAGAAGTACCTGTGGTGGAAGAGATAC 720
N  T  F  V  H  I  V  M  Y  S  Y  Y  G  L  A  A  L  G  P  H  M  Q  K  Y  L  W  W  K  R  Y  203
CTCACCTCTCTGCAGCTGGTGCAGTTTGTGCTGTTCCTCCTGCACACGGGCTACAACCTGTTCACAGAGTGCGACTTCCCCGACTCCATG 810
L  T  S  L  Q  L  V  Q  F  V  L  F  L  L  H  T  G  Y  N  L  F  T  E  C  D  F  P  D  S  M  233
AACGTGGTCGTGTTTGGTTACTGCGTCACCCTCATCATCCTCTTCAGTAACTTCTATTTTCAGAGCTACCTCAACAAGAAGAAGCAGAAA 900
N  V  V  V  F  G  Y  C  V  T  L  I  I  L  F  S  N  F  Y  F  Q  S  Y  L  N  K  K  K  Q  K  263
TAAGACACAGTGATTGTGGAGCTTTATGAAACTGTGAACAGCCGGAAAGAACACACCCCTAAGGATGGAGAAGGCAGAAGCACACTGCCG 990
-
CGATGAATGAGAAGAATCCAGAGACCCTCAGCAGAAAGTTCTCCCTCAGCTCCTTCAACACCGATGAAAAGGAATCTTGATGGTACTGTG 1080
TGTGAAAATCCTACACGGTGCCCTCTCATGCACATGTGCCTTACTGTCAAAGCATCACAGTGTGTTCCTATGTTAAATGATTGTTATGGT 1170
CCATTCACACAAAATAAAAATACCATCAATTTGAAAAAAAAAAAAAAAAAAAAAAAAA                                 1228
```

Figure S5. Nucleotide and deduced amino acid sequence of Elovl8 in hybrid grouper (GenBank accession number MZ713364). The nucleotide sequence is numbered from the first base at 5′ end, started in codon (ATG) and stopped in codon (TGA). The putative transmembrane regions are in gray; the putative histidine-richdomain (HXXHH) is in bold; the predicted seven (I-VII) putative membrane-spanning domains are underlined; the ER retrieval signal is wavy underlined.

ATAGGGGAATGAGTGGGTGAATGAGTCCGTGAACATATTAGACAAGACAAGAGGATGAATTTTGGGATGTTTACTATTGGAAATGACTGT 90
GACCGGAGCGGGAGCAGATAAAATGAGTCGGTGTGTCAGTGTGACTGAAACCAAGGGAGAGTAGCCAAAATCTGGATACTGTGTCAAACT 180
TTGTGGCTGACTGGCCCTTTAGTGTTCAGGTGGACCCAGGCCAGAGACAGCAGTGAGGATGGGAGGTGGAGGCCAGCTGACGGAGCCAGG 270
M G G G G Q L T E P G 11
GGAGCAGAGCAGTGGGCGGGCTGGTGGTGTCTACACCTGGGAGGAGGTGCAGAGTCACTGCGACAGGAATGACCAGTGGCTGGTGATCGA 360
E Q S S G R A G G V Y T W E E V Q S H C D R N D Q W L V I D 41
TCGGAAGGTTTACAACATCACACAGTGGGCTAAAAGGCACCCAGGAGGGATCCGGGTCATCAGCCACTACGCTGGAGAGGATGCCACGGA 450
R K V Y N I T Q W A K R H P G G I R V I S H Y A G E D A T E 71
GGCGTTCACCGCTTTTCATCCTGATTTAAAGTTTGTGCAAAAGTTTCTGAAGCCCCTGCTGATTGGAGAGCTGGCAGCGACAGAGCCCAG 540
A F T A F H P D L K F V Q K F L K P L L I G E L A A T E P S 101
CCAGGACCGAGACAAAAACGCAACAATCATACAAGATTTCCACACTTTACGGGTGCAAGCGGAGAGGGAGGGTTTGTTTCAAGCTCGGCC 630
Q D R D K N A T I I Q D F H T L R V Q A E R E G L F Q A R P 131
TTTGTTCTTCTGCCTCCACCTGGGTCACATCCTGCTGCTGGAGGCCCTCGCCTGGCTCATCATCTGGGTCTGGGGAACCAGCTGGACGCT 720
L F F C L H L G H I L L L E A L A W L I I W V W G T S W T L 161
GACGTTTCTGATCTCCTTCATGCTGGCGACCGCTCAGTTGCAGGCTGGATGGCTGCAGCACGACTTTGGCCACCTGTCTGTCTTCAAGAA 810
T F L I S F M L A T A Q L Q A G W L Q **H D F G H** L S V F K K 191
GTCCAGCTGGAATCACATATTGCACAAGTTTGCCATCGGCCATTTGAAGGGAGCGTCTGCCAACTGGTGGAATCACCGGCATTTCCAGCA 900
S S W N H I L H K F A I G H L K G A S A N W W N H R **H F Q H** 221
TCACGCTAAACCCAACATCTTCAGTAAGGACCCTGATGTCAACATGTTGCACATCTTTGTAGTTGGAACCACTCAACCAGTGGAGTACGG 990
H A K P N I F S K D P D V N M L H I F V V G T T Q P V E Y G 251
CATAAAGAAGATTAAATATATGCCCTATCATCACCAACACCAGTACTTCTTTCTTGTTGGACCACCGCTGCTCATTCCAGTCTACTTCCA 1080
I K K I K Y M P Y H H Q H Q Y F F L V G P P L L I P V Y F H 281
CATTCAGATAATACGCACCATGATTTCCCGCCATGACTGGGTGGATCTGGCTTGGTCCATGTCTTACTACCTGCGCTACCTGTGCTGTTA 1170
I Q I I R T M I S R H D W V D L A W S M S Y Y L R Y L C C Y 311
CATTCCCATGTATGGCCTGCTTGGCTCAGTGTTGCTCATCAGCTTTGTCAGGTTTTTGGAGAGTCATTGGTTTGTGTGGGTGACTCAGAT 1260
I P M Y G L L G S V L L I S F V R F L E S H W F V W V T Q M 341
GAATCATCTACCAATGGACATCGACCACGAGAAGCACCGGGACTGGCTGTCCATGCAGCTACAAGCCACCTGTAACATCAAACAATCCCC 1350
N H L P M D I D H E K H R D W L S M Q L Q A T C N I K Q S P 371
CTTCAATGACTGGTTCAGTGGACACCTCAACTTTCAAATCGAACACCATTTGTTTCCCACGATGCCGCGCCACAACTACCACCTGGTGGC 1440
F N D W F S G H L N F **Q I E H H** L F P T M P R H N Y H L V A 401
CCCGCTGGTCCGTGCACTGTGTGAGAAACATGGGATTCCATACCAGGTGAAAACACTGTGGCGAGGCATCACTGATGTTTTCATCTCATT 1530
P L V R A L C E K H G I P Y Q V K T L W R G I T D V F I S L 431
GAAGAACTCAGGTGACCTCTGGCTCGACGCATATCTCCATAAATGACCAGTTTTATTCTCGACACGACAAGGAGTGATGTTGTTTCTCCT 1620
K N S G D L W L D A Y L H K - 445
CTTTTGCATCATAAACTGATTGTATCTGTTTGGTTTTATAATTCAGTTGATAGTGTGGGAATGATCTTTTCTTAGCTCCGGTGTTATAGT 1710
TCAGTCTTTTTTAGATTCTGTGCCGGGTTTTTAGTGCTCACAGGATTTCCCTGGTTGCCTTACAGTGTCTTGAACAACTGTAGTATGGTG 1800
TTGTAGGTGCAGCTGTGAAACTGATGTGAGGTATTTAAAGTGATGTTTTTTCATTATCTATCATGAACAATAAAATATGACAAATCCTGT 1890
ATAACTGAGTATTAGTTATAGTTAATTTAACTTCTTTACTTGCAACTCTTGTTCTGCTCTGCTTCTGTTTAAATAAAGGAACTGTATAAA 1980
AAAAAAAAAAAAAAAAAAAAAAAAAA 2006

Figure S6. Nucleotide and deduced amino acid sequence of Fads2 in hybrid grouper (GenBank accession number MZ713366). The nucleotide sequence is numbered from the first base at the 5′ end, started in codon (ATG) and stopped in codon (TGA). The Cytochrome b5-like Heme/Steroid binding domain is in gray; the putative histidine-rich domain (HXXHH, HDXGH and QXXHH) is in bold; the predicted four (I-IV) putative membrane-spanning domains are underlined.
